# Marangoni Flow‐Driven Angular Self‐Assembly of Cellulose Nanocrystals: The Tale of Tilted Tactoids and Folded Domains

**DOI:** 10.1002/smtd.202401966

**Published:** 2025-05-19

**Authors:** Yuchen Zhu, Tadeusz Balcerowski, Ahu Gümrah Dumanli

**Affiliations:** ^1^ Department of Materials The University of Manchester Oxford Rd Manchester M13 9PL UK; ^2^ Henry Royce Institute The University of Manchester Oxford Rd Manchester M13 9PL UK

**Keywords:** angular deposition, cellulose nanocrystals, marangoni flow, tactoid domains

## Abstract

Cellulose nanocrystals (CNCs) can spontaneously self‐assemble into cholesteric photonic films with vibrant colors with multidomain structures and variations in cholesteric pitch. Herein, an angular deposition technique is employed to harness capillary and Marangoni flows to fabricate CNC photonic films with spatially tunable structural colors spanning from red to blue. A crucial relation between the substrate angle, the development of color zones, film coverage and film thickness is discovered. The color range of the photonic films can be shifted by tuning the size distribution of CNC particles is also demonstrated. As the CNC particles and tactoids are deposited on the substrate, a central deformation line emerged with tilted and folded domains, which is a consequence of Marangoni flow‐induced deformation of the tactoids at the early stages of deposition. Further in the process, well‐aligned domains emerged at the bottom of the substrates, indicating the simultaneous kinetic onset of multiple gelation processes which depend on size segregation across different color zones. Such insights allow us to tune the color domains using angular deposition and manipulate the kinetic arrest phase transition to produce more uniform and homogeneous films.

## Graphical abstract

Cellulose nanocrystals spontaneously self‐assemble into cholesteric structures through a new pathway using angular deposition. This work explores the film formation dynamics and chiroptical properties emerging due to tilted and folded domains, due to Capillary and Marangoni flows.

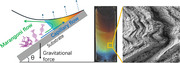

## 1 Introduction

Cellulose nanocrystals (CNCs) are highly polydisperse rod‐like colloidal particles with high aspect ratios (12–20).^[^ ^1^, ^2^, ^3^, ^4^, ^5^ ^]^ Due to the acid hydrolysis method used in the isolation of CNCs, they possess half‐sulphate ester groups providing electrostatic stability and contributing to their structural organization.^[^ ^6^ ^]^ In water suspensions, upon evaporation, the CNCs can spontaneously self‐assemble into cholesteric mesophases through a combination of attractive and repulsive colloidal interactions.^[^ ^6^, ^7^, ^8^ ^]^ The cholesteric structure can be preserved in the solid form after complete evaporation of water.^[^ ^9^, ^10^, ^11^ ^]^ These photonic films demonstrate remarkable optical properties, e.g., strong birefringence, circular dichroism, and selective reflection, making them attractive for various applications in colorimetric sensors, anti‐counterfeit materials, and colorful coatings.^[^ ^2^, ^4^, ^5^, ^12^, ^13^ ^]^

The most commonly reported way of fabricating photonic CNC films is via drop‐casting.^[^ ^14^, ^15^, ^16^ ^]^ While drop‐casting offers a facile method to produce these films, it often results in structural and optical artefacts due to the sessile droplet geometry, flow dynamics and non‐equilibrium effects.^[^ ^14^, ^17^, ^18^ ^]^ Moreover, the mechanism behind the formation of structural defects, e.g., misaligned and tilted cholesteric structure, and the optical signature of these defects remains poorly understood. In this study, we utilized angular deposition to precisely control the structural artefacts by tuning the flow dynamics during the self‐assembly process.

In the drop‐casting configuration starting from a dilute suspension, initially, CNCs are suspended isotropically in water.^[^ ^9^, ^19^ ^]^ As the water evaporation progresses, the increase in the CNC concentration leads to the formation of a gel phase, i.e. the liquid becomes viscous.^[^ ^20^, ^21^, ^22^ ^]^ At this stage, the suspension consists of cholesteric nucleation domains (known as tactoids), suspended CNCs as well as different‐sized particle clusters.^[^ ^23^, ^24^, ^25^, ^26^ ^]^ As the evaporation progresses further, the suspension phase will change from biphasic to fully cholesteric, i.e., cholesteric domains will no longer be segregated by isotropic suspension.^[^ ^27^, ^28^ ^]^ Instead, the different domains will form grain boundaries with relatively complex cholesteric reorganization and topological defects.^[^ ^9^ ^]^ With the merger of the cholesteric phases at the later stages of the self‐assembly, the gel‐like suspension becomes kinetically arrested.^[^ ^24^ ^]^ In this trapped state, the further evaporation of the remaining water will cause mechanical compression of the cholesteric structure.^[^ ^3^, ^5^ ^]^ This compression can cause the tactoids to distort and reduce their tilt angle, leading to a more compact structure.^[^ ^29^, ^30^, ^31^, ^32^ ^]^ By contrast, alternative approaches, e.g., applying a magnetic field or employing 3D printing, can yield uniform ordering of CNC tactoids.^[^ ^33^, ^34^ ^]^ In addition, introducing polymer dopants into the CNC suspension allows for tuning the alignment and orientation of CNC tactoids.^[^ ^35^ ^]^

The previous work laid the foundations for achieving uniform coloration and scaling up. Nevertheless, insights into the fine interplay between the Marangoni flow, capillary flow and gravitational settling are limited in regard to the formation of tactoids, their alignment and gelation mechanisms. Our work addresses this critical gap by producing, for the first time, angularly deposited photonic CNC films. This method not only mitigates artefacts commonly associated with drop casting but also establishes a delicate equilibrium among capillary forces, Marangoni effects, and gravitational influences. Notably, we demonstrate that adjusting the deposition angle modulates the meniscus at the deposition interface, thereby dictating the structural coloration of the resulting films. By utilizing angular deposition, we open new avenues for scaling up the production of photonic CNC films and gain deeper insights into the complex flow mechanisms involved.

## 2 Results and Discussion

### 2.1 Drop‐Casted CNC Films and the Flow‐Determined Tilted Domains

**Figure 1:**
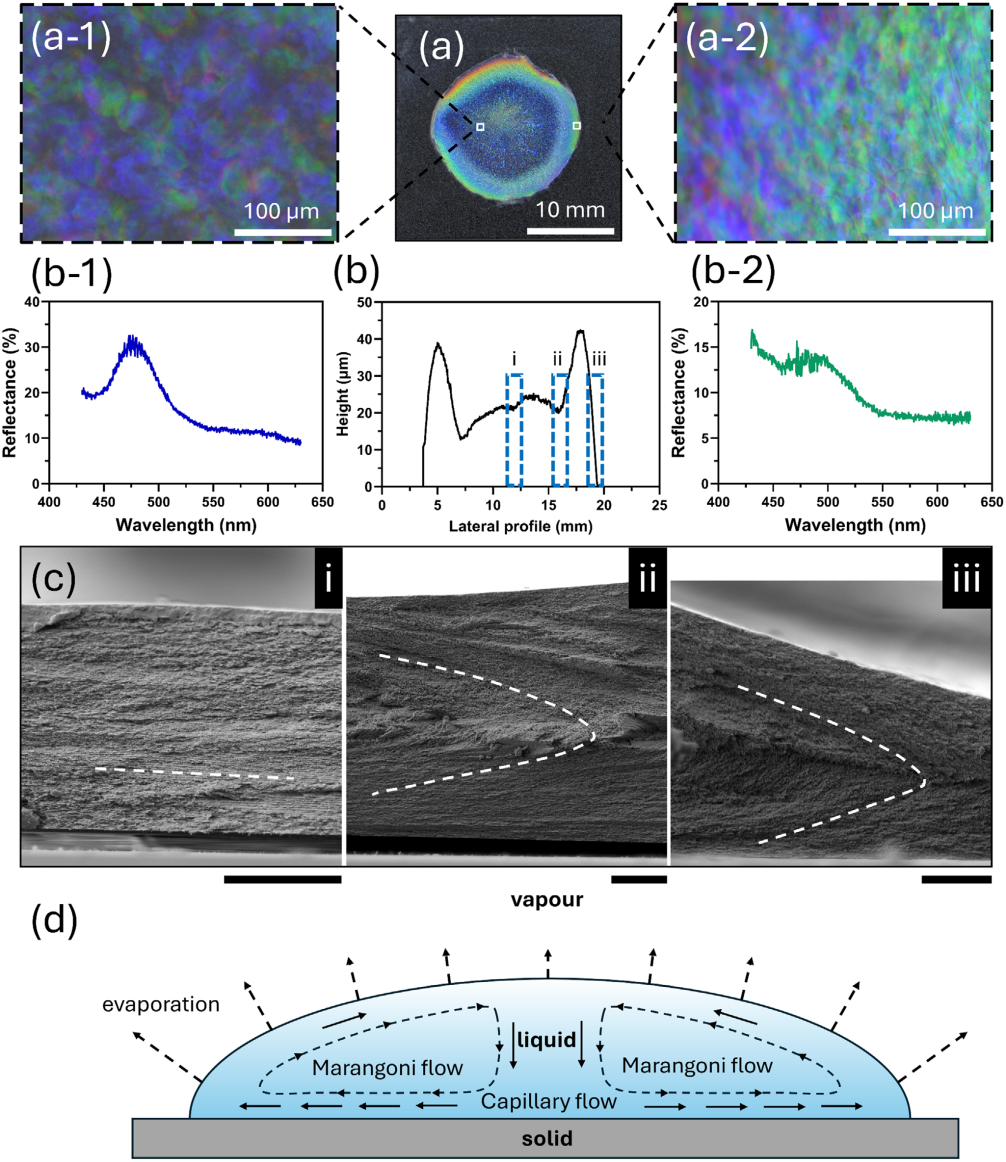
Drop‐casted CNC films. a) The digital photograph of the drop‐casted CNC film and (a‐1, a‐2) POM images of the edge and the middle of the CNC film under cross‐polarizers. b) The lateral profilometry of the CNC film and (b‐1, b‐2) the reflectance spectra of the CNC film taken from the positions of images (a‐1) and (a‐2). c) Cross‐sectional SEM images taken from the different test points (i central region, ii and iii on both edge sides of coffee ring band) on the film, showing the distinct orientation and alignment of CNCs. d) Schematic diagram of flow dynamics within the droplet dominated by capillary and Marangoni flow. The scale bar in the SEM images is 10 µm.

**Figure 2:**
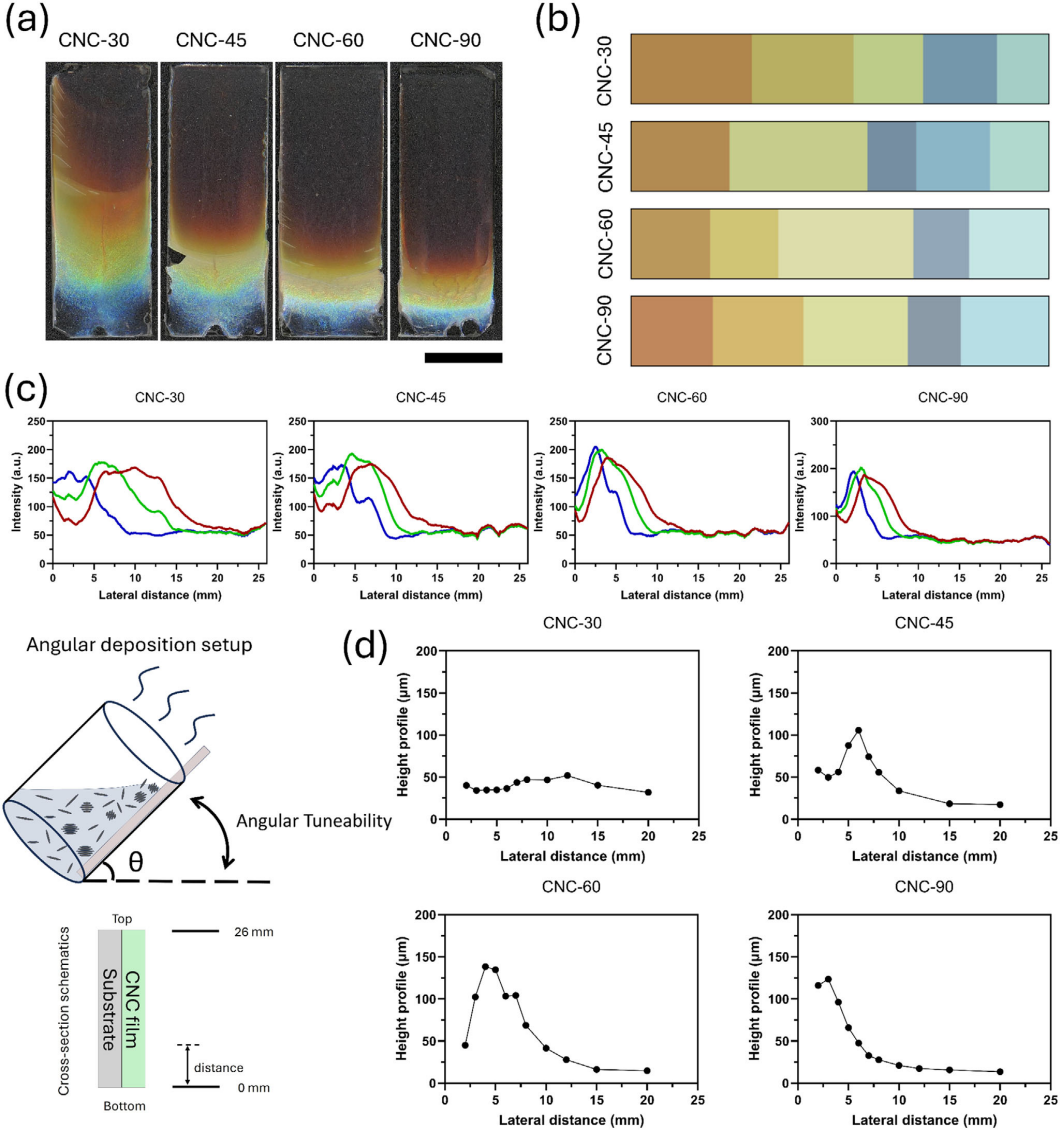
Angularly self‐assembled CNC films. a) Digital photographs of CNC films imaged under the normal incidence of light and the films were prepared at angular set‐ups at 30°, 45°, 60°, and 90°; b) Color analysis on the CNC films using K‐means fitting algorithm, c) RGB color analysis on acquired CNC films, derived from the digital bulk images displaying the relative ratios of the color domain coverage, i.e. integrated area under red gets smaller as the deposition angle is moved from 30 degrees to 90 degrees; d) Height profile analysis on CNC films cleaved along the longitudinal direction. The scale bar is 7 mm.

To establish a baseline for the optical properties of the CNCs, we fabricated photonic films via the drop‐casting method. **Figure** **1**(a) shows the formation of the coffee ring pattern on the glass substrate after complete evaporation and the emergence of a rainbow band. The polarized optical microscopy (POM) images in Figure 1a‐1,a‐2 demonstrate a visual color shift from blue to green moving from the central region to the peripheral edge. This is consistent with the reflectance analysis at the centre and edge points, as shown in Figure 1b‐1,b‐2. Cross‐sectional SEM images of the coffee ring pattern were taken at points i, ii and iii, represented in Figure 1,b,c. In Figure 1c,i, the film centre presents with a uniform coloration and aligned domains. In contrast, a dense ring of CNCs was observed at the periphery, exhibiting a rainbow coloration and tilted domains, as illustrated in Figure 1c,ii,iii. The unique misoriented nanostructure results from the combined effects of capillary and Marangoni flows, which influence the alignment of CNCs relative to the substrate during the migration toward the drying front. In drop‐casting, the capillary flow in Figure 1d arises due to the differential evaporation rates across the droplet's surface.^[^ ^14^, ^36^ ^]^ Such inhomogeneous evaporation creates a radial outward capillary flow carrying the suspended CNCs and tactoids to the edge of the droplet for particle deposition.^[^ ^37^ ^]^ Meanwhile, the Marangoni flow is induced within the droplet, due to surface tension gradients caused by evaporation‐induced concentration and temperature differences across the liquid‐air interface and partially suppresses the coffee ring effect.^[^ ^14^ ^]^ Moreover, Chang et al. examined the effect of temperature on the color distribution of CNC films and revealed that the Marangoni flow induced by elevated temperature results in a more dispersed coloration.^[^ ^38^ ^]^ Further evaporation of water kinetically arrests these titled domains at the liquid‐substrate interface during the gelation process. Simultaneously, at the liquid‐air interface, the Marangoni flow transports the tactoids back to the central region, where they manifest as a circulatory pattern causing a distinctive domain fold in the final structure. Figures S1 and S2 (Supporting Information) show the size distribution of extracted CNC particles and validate the left‐handed helical structure of CNC films after self‐assembly.

The presence of distinct tactoid domains across the drop‐casted CNC film raises the question of whether the alignment of domains and the resulting optical properties can be tuned by switching to the angular deposition setup. To quantitatively assess the impact of deposition angle on domain alignment, we fabricated CNC photonic films using an angular deposition setup.

### 2.2 Angular Deposition of the CNCs‐ Color Separation and Domain Tilting

The angular deposition of the CNC films was achieved by inclining the container at a defined angle relative to the base plane and those samples are labeled with CNC‐θ, θ representing the inclined angle, as shown in **Figure** **2**. In Figure S3 (Supporting Information), we present the initial still images of the angular deposition set‐ups and the contact angles between the meniscus and the substrates calculated using image analysis techniques. Compared to the drop‐casted films, all CNC‐θ films demonstrated clear distinctive differences in their visual appearance, such as the dominating color appearance and the way the coffee ring manifests in the films. For example, while the drop‐casted films had a blue center that was considered as the uniformly assembled region, the angular deposited films had mainly redshifted coloration. It is worth mentioning that the uniform color domains and their color proportions of the photonic CNC films varied drastically in the angular deposition setup. Overall, the largest deposition sequence was achieved at 30° with a film color gradient starting from red and transitioning into orange, yellow, green, and blue, Figure 2a.

To provide a more quantitative analysis of the color appearance in these samples, the digital photographs shown in Figure 2a were analyzed by a K‐means fitting algorithm. The algorithm was applied to the colored regions exclusively, and the photographs were processed into 2‐D bars, as shown in Figure 2b. From this quantitative analysis, the area of the samples exhibiting color was calculated; interestingly, the area of the colored photonic film decreased significantly with the deposition angle. At the same time, the distribution of the colors, i.e. the color fraction (*f*) ratio *f*_red_: *f*_green_, did not vary to such an extent. The sample CNC‐45 deviated from the observed trend as the red portion of the color region (*f*_red_) was observed to be contracted, with little compression on the blue zone, leading to a larger blue ratio (*f*_blue_). The alteration in the color distribution could be an indication of the change in the force balance at the liquid‐solid interface. Flow dynamics modeling at different deposition angles and direct observation at the interface using particles at several micro length scales could provide further insights into the deposition rate, color distribution, and film coverage. Following the CNC film coverage analysis, the photographs were decomposed into their red, green, and blue (RGB) channels, as depicted in Figure 2c. The color information was extracted from micrographs using ImageJ and the RGB data was extracted and averaged from each micrograph using K‐Means clustering and plotted onto a 2‐d chart. Finally, ImageJ was used to quantitatively analyze the contribution of the RGB channel to the overall color appearance of CNC film and normalized against the proportion of the colored area within the CNC film. The RGB analysis allowed us to assess the contribution of each channel to the overall coloration of the CNC films and the influence of the deposition angle on the distribution and intensity of the color. The CNC‐30 exhibited the most extensive color region, with each RGB channel covering an area of 20 mm. As the deposition angle increased, this color region gradually diminished, reaching a minimum coverage of 10 mm at a 90° angle.

Such color changes in the film coverage also influenced their thickness profile, as shown in Figure 2d. Cross‐sectional SEM analysis revealed a strong dependency of film thickness on the inclined angle. As the inclined angle increased from 30° to 90°, the film exhibits nonuniform thickness, with a significantly thicker layer near the bottom edge compared to the rest of the surface. The film thickness gradient indicates the accumulation of a large number of particles during the later stages of evaporation. In the angular setup, the curvature of liquid–air interface varies with the inclination angle. At lower angles, the interface of the meniscus region,^[^ ^39^ ^]^ where the solvent and solid substrate are in contact, is more extended, which promotes the migration of CNC particles toward the meniscus edge by enhancing capillary forces.^[^ ^40^ ^]^ Yet, the slope angle can also minimize the formation of a coffee ring pattern by moderating this particle migration because of the gravitational field, which leads to a film with consistent thickness. At a deposition angle of 30°, capillary forces effectively counteract gravitational forces, enabling cholesteric thin film to accumulate at the meniscus interface due to the low contact angle (Figure S3c, Supporting Information). As the angle increases to 90°, the higher water contact angle diminishes the effectiveness of capillary forces in drawing particles toward the substrate. This results in a higher concentration of the solution at the lower region, leading to delayed film deposition after gelation during self‐assembly and the formation of a thicker layer of CNC films.

The thickness profile of the CNC films is directly related to the local concentration of CNC particles at the meniscus region for film deposition, which in turn influences its optical properties. The thickest point of the film, representing the highest concentration of CNCs, should correspond to the smallest pitch of CNC tactoids,^[^ ^41^ ^]^ reflecting the shortest wavelengths in the color pattern. However, in our study, the thickest point of the film did not display the expected shortest reflection wavelength within the observed color pattern (CNC‐30 showing yellow at 8 mm, CNC‐45 exhibiting green at 6 mm, CNC‐60 demonstrating green at 4 mm, CNC‐90 showing green at 3 mm). This finding indicates that the pitch of cholesteric films may be influenced by other factors rather than solely influenced by CNC concentration.

To better understand the impact of CNC size distribution on the color gradient of the resulting photonic films, we utilized three batches of CNCs with varying length distribution and aspect ratio for the angular deposition. Figure S4 (Supporting Information) shows that the color appearance of photonic films assembled from longer CNCs with a large aspect ratio exhibits a blue‐shifted color pattern, with an absence of red hues. By contrast, films made from shorter CNCs with a small aspect ratio display red‐shifted coloration. The change in the color is attributed to the higher twisting power of the higher aspect ratio particles, which directly reduces the pitch.^[^ ^42^, ^43^ ^]^ Therefore, the size distribution of CNCs also significantly influences the color appearance of the CNC films produced using the angular deposition method.

**Figure 3:**
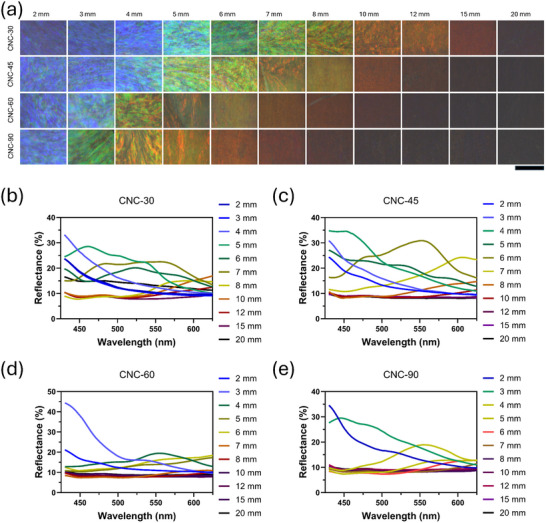
High‐resolution POM images of CNC and their reflection spectrum. a) Reflection polarized optical photographs of CNC films imaged under normal light at different analysis points and the corresponding UV–vis spectroscopy of b) CNC‐30, c) CNC‐45, d) CNC‐60, and e) CNC‐90. The scale bar is 200 µm.

To quantify the effect of deposition angle on the color distribution in the CNC films, we captured a series of POM sample images at various analysis points and stitched them into a comprehensive color map in **Figure** **3**a. From the color mapping, the region of gradient color had the largest coverage in CNC‐30, while CNC‐90 exhibited the smallest color coverage. It is observed that each sample exhibited a color transition region, characterized by a smooth gradient of colors, shifting seamlessly from one hue to another, as shown in CNC‐30 at 5 mm, CNC‐45 at 4 mm, CNC‐60 at 3 mm, and CNC‐90 at 2 mm. More specifically, the color transition from blue to green shifts linearly downward toward the bottom region as the inclined angle increases. This gradient change was also observed in the color transition between the red and colorless. When the analysis point was fixed at 5 mm, a clear redshift in color was observed across the inclined samples from 30° to 90°. At 10 mm, the color transition followed this redshift pattern, shifting from orange to red and then to colorless. By 20 mm, all samples, regardless of the inclined angle, appeared dark under POM. This color gradient distribution was experimentally corroborated by UV–vis spectroscopy, as shown in Figure 3b–e. Instead of reflecting a single narrow band of wavelengths, the CNC film reflects a broader range of wavelengths, resulting in a less sharp and more diffusive color. This effect is particularly evident in the blue‐green transition region (CNC‐30 at 5 mm, CNC‐45 at 4 mm, CNC‐60 at 3 mm, and CNC‐90 at 2 mm), where UV–vis analysis shows broad reflection peaks spanning from 450 to 550 nm. In contrast, each sample displayed a redshifted pattern as it progressed toward the top region and a final transparent film at the top edge. Additionally, the pitch analysis of color zones on the CNC films at corresponding analysis points illustrated in Figure S5 (Supporting Information), showed a consistent redshift with an increase in pitch value.

### 2.3 Quantifying the Effect of Deposition Angle on the Domain Transition

**Figure 4:**
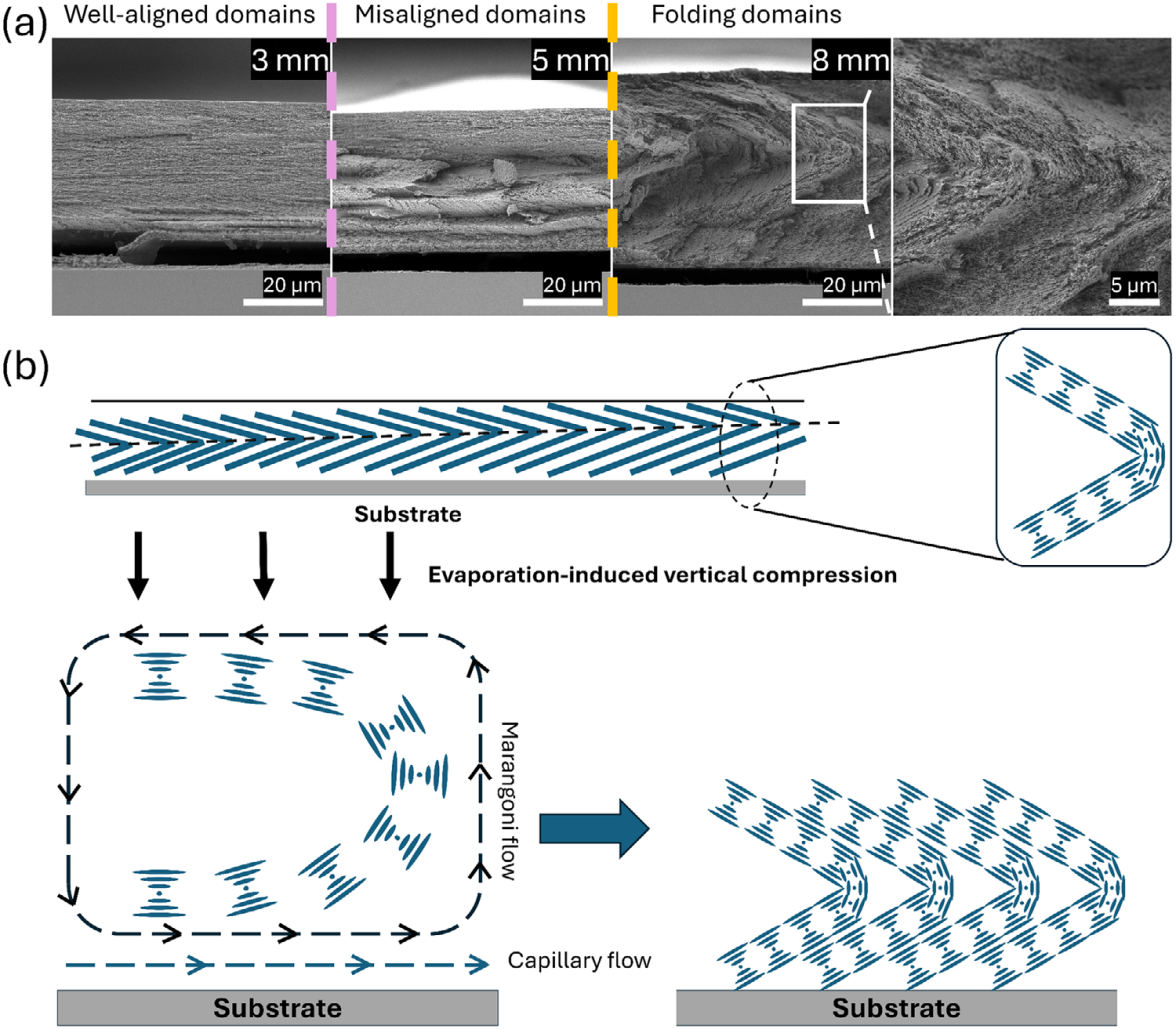
Lateral structural transition of CNC tactoid domains. a) Representative SEM images of multi‐hierarchical microstructure of CNC tactoid domains at different structural transition stages and b) Schematic diagram for the formation of CNC folding domains.

To further elucidate the microstructural characteristics of the CNC films produced via angular deposition, the samples were cleaved in half along the longitudinal direction to expose their cross‐section. The microstructural assessment in **Figure** **4**a revealed three distinct regions: domains with folded tactoids, misaligned domains with a defect line, and well‐aligned domains near the bottom edge of the glass substrates. The formation of those domains is a result of the film deposition process, with the sequence starting from folding domains, transitioning into misaligned domains, and finally evolved into well‐aligned domains.

Figure S6 (Supporting Information) showed that at larger deposition angles, the fraction of the folded domains concerning the total area of the film accounted for a greater proportion compared to smaller deposition angles. Interestingly, the folding domains terminate at the thickest point of the film for each sample, specifically at 8 mm for CNC‐30, 6 mm for CNC‐45, 5 mm for CNC‐60, and 3 mm for CNC‐90. In the region with folding domains, the deposition rate was estimated to be fastest causing the tactoids to deform under the Marangoni stresses.^[^ ^18^ ^]^ In this area, we identified a folding ratio, i.e. ratio of folding point to the thickness of the film, to correlate the deposition angle and folding of the film, as depicted in Figure S7a,b (Supporting Information) shows that the folding ratio remains constant at ≈0.7, regardless of the deposition angle and the test location. Moreover, the transition from the folding of the domains to misaligned domain structures exhibited an angle‐dependent feature as shown in Figure S7c (Supporting Information).

We propose that the flow dynamics in the meniscus region alter the interplay between the capillary and Marangoni forces as well as the gelation process induced by evaporation. This alteration in the dominant forces affecting the particle flow and agglomeration leads to the transformation of these distinct domains of tactoids with different pitches in terms of their alignment in the deposited film. As the CNC particles and tactoids approach the meniscus, the Marangoni flows cause the tactoids to tilt at an angle relative to the flow direction.^[^ ^44^ ^]^ This folding line feature was kinetically arrested as the system approached the gelation point, as shown in Figure 4b. In the misaligned domains, the influence of Marangoni flows is minimal and competes with gravitational forces, leading to the random orientation of the tactoids. The well‐aligned domains are formed at the bottom edge of the glass substrate at the later stages of self‐assembly. In this stage, the CNC suspension is in a gel form and evaporates more slowly. These two effects combined with the counter‐balanced capillary and Marangoni flows, cause strong vertical alignment of tactoids by anchoring them at the liquid‐substrate interfaces. After kinetic arrest, further evaporation induces vertical compression on the tilted tactoids, leading to a variation in pitch with respect to the tilt angle.

Furthermore, the tilt introduces optical anisotropy due to the light‐domain interaction at different angles. This can lead to different circular dichroism (CD) signals, providing insight into the 3D arrangement of the CNCs.

### 2.4 Tilted Domains Manipulating the Reflection Spectrum and Circular Polarization state

**Figure 5:**
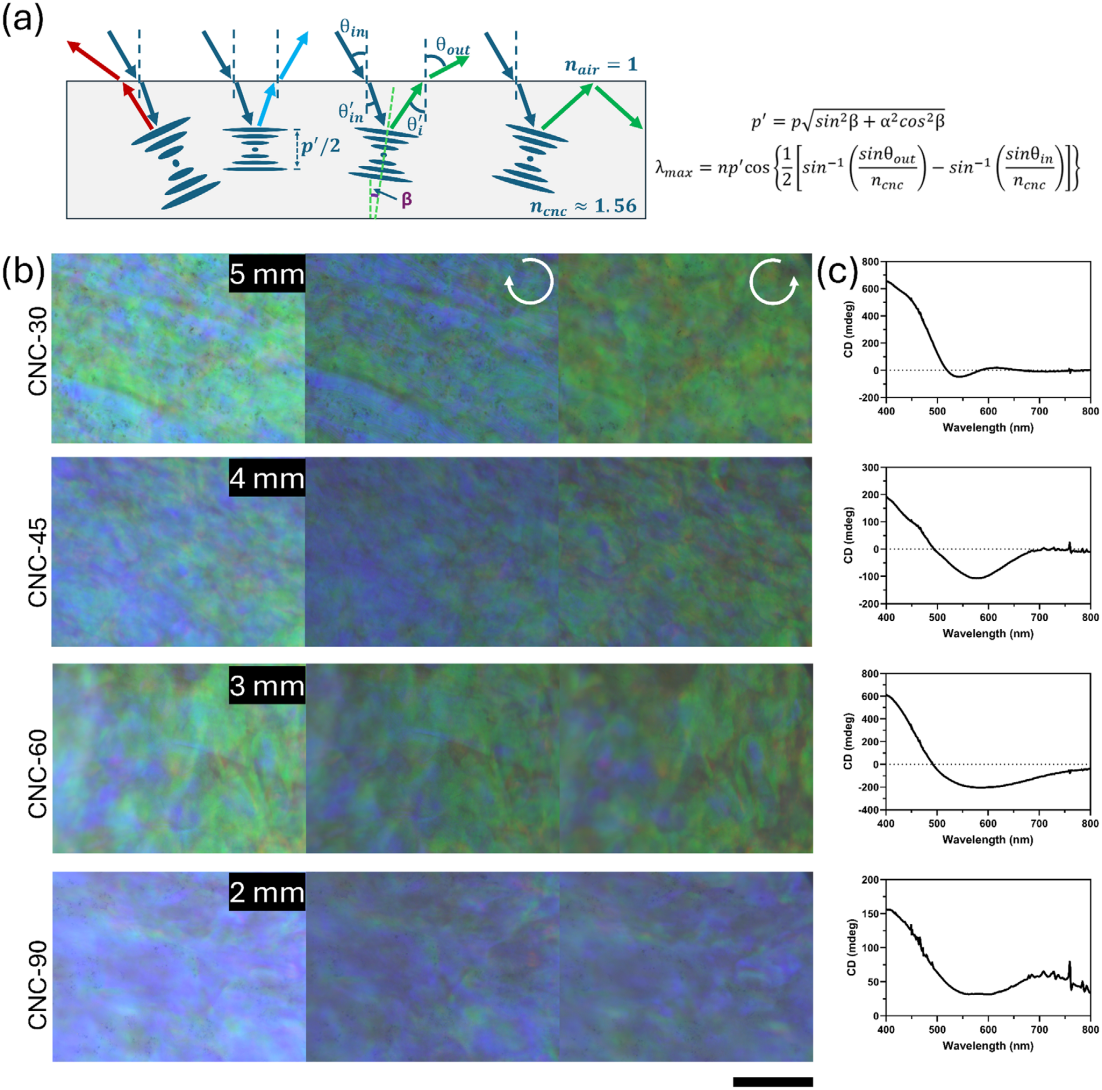
Digital photographs of CNC films at their multidomain region under circularly polarized microscopy and their resulting CD spectroscopy. a) Schematic diagram of the interaction of the tilted domains with incident light and the formulation for the reflective photonic bandgap. b) Circularly polarized microscopy of the tactoid domains at their transition region. c) CD spectroscopy analysis on the misaligned domain region of CNC films. The scale bar is 100 µm.

The microstructural analysis of the deposited films along the longitudinal axis through examining the cross‐section revealed the presence of a domain transition region from misaligned and folded domains. Such transition leads to unique optical features within the domain, such as the circular dichroism rather than circular on‐off states as expected from left‐handed cholesteric structures. In these misaligned domains, CNC tactoids orientate themselves at varying angles, denoted as β, leading to different pitches under vertical compression, as shown in **Figure** **5**a. This variation explains the mixed colors observed in the transition region of the CNC film. Herein, we select each sample of misaligned domains (CNC‐30 at 5 mm, CNC‐45 at 4 mm, CNC‐60 at 3 mm, and CNC‐90 at 2 mm) for studying their chiroptical properties. Figure 5b shows that CNC‐30 and CNC‐45 in particular exhibit circular dichroism. Their color shift from a mixed blue‐green to pure green upon switching light from LCP to RCP, but this shift is less pronounced in CNC‐60 and CNC‐90. The tilted and folded domains at the early stages of the film deposition cause elliptical polarization of light rather than being purely left‐handed circularly polarized.^[^ ^30^ ^]^ For the uniform areas using the modified Bragg equation, the wavelength is calculated at 405.6 nm for CNC‐30 at 5 mm, 458.9 nm for CNC‐45 at 4 mm, 436.8 nm for CNC‐60 at 3 mm, and 405.6 nm for CNC‐90 at 2 mm. The cholesteric organization and circular dichroism of the CNC films were also confirmed via CD spectral analysis, confirming their preferential reflection of the left‐handed circularly polarized light.^[^ ^3^, ^45^ ^]^ Interestingly, the CD spectra of these misaligned domains revealed that each sample, except for CNC‐90, exhibits both positive and negative peaks at different wavelengths; a strong positive peak appears ≈400 nm consistent with the blue reflection of the films from their polarized optical microscopy analysis. This strong peak is followed by a broad and weak negative dip implying right‐handed circular polarization between 500 and 600 nm (green to orange in the spectra), Figure 5c. If the cholesteric domains of the CNC films were perfectly aligned against the glass substrate's normal incidence, the films would have exclusive left‐handed CD signal without any contribution from negative CD signal. However, the presence of the tilted and folded domains induces deformation in the left‐handed cholesteric arrangement and contributes to phase retardation. This effect is particularly significant in other regions of CNC films produced using the angular deposition setup, as shown in Figures S10–S13 (Supporting Information). Therefore, the appearance of both left and right CD signals is most likely attributable to these domains. Similar observations (presence of both LCP and RCP) were recorded in both CNC and HPC‐based solid films previously, where cholesteric order was distorted due to the self‐assembly kinetics and manufacturing method.^[^ ^29^, ^30^, ^31^, ^46^ ^]^ We also account that the optical assembly of the instrument may cause signal artefacts (particularly in solid films), as well as the presence of linear dichroism of the CNCs, may have contributed to the mixed polarization state.^[^ ^47^, ^48^ ^]^

In the transition region, we observed that the domains are tilted in random directions with varying angles, as observed from the SEM cross‐sectional images, Figure S5 (Supporting Information), and schematic diagrams of Figure 5a and Figure S8 (Supporting Information). Further compression of the film during the late stages of the evaporation process alters the periodicity of the helicoidal structures along the helix axis, which results in an angle‐dependent pitch change in the compressed structure,^[^ ^31^ ^]^ as described in Equation (1) (Supporting Information). By analyzing the folding domains of CNC tactoids in Figure S9 (Supporting Information), we have concluded that domains with steeper tilt angles result in a larger pitch once they are completely evaporated. This is due to the presence of the tilt angle, which causes the compression force to divide into its 3D vector components, reducing the effectiveness of the direct compressive force on a tilted domain. Consequently, the wavelength of the reflected light will red‐shift, as shown in the equation in Figure 5a, resulting in different colors appearing in tilted domains compared to nontilted regions.

### 2.5 Gravity‐Driven Size Gradient Distribution of CNC Tactoids Leading to Distinct Color Zones

**Figure 6:**
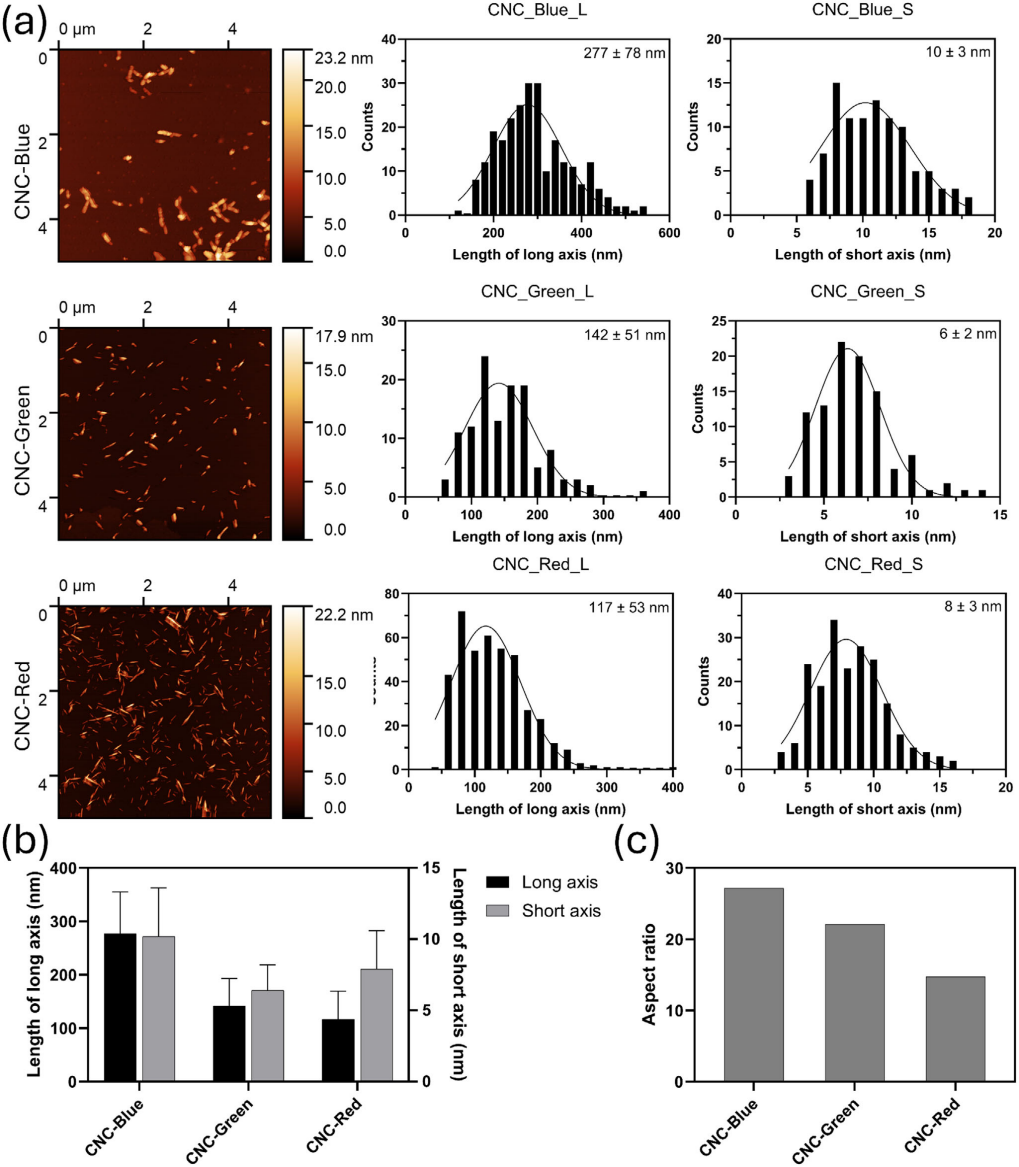
Size distribution analysis of CNC films flaked from each color zone. a) AFM images of CNCs from blue film pieces (CNC‐Blue), from green film pieces (CNC‐Green), and from the red film pieces (CNC‐Red) and b) Size distribution analysis on those images for length determination of long axis and short axis, c) Mean aspect ratio analysis on the extracted CNC particles.

While these tilted tactoids with varying pitches partially explain the broader color distribution across the film in Figure 3, the formation of gradient color zones is quite an intriguing concept. Among all colloidal systems CNCs show a high polydispersity in terms of their size distribution and electrostatic interactions. Despite their polydispersity, CNCs still tend to form highly organized photonic structures. This is due to the strong particle‐particle interactions in the longitudinal axis, which leads to nematic and twisted conformations.^[^ ^23^ ^]^ We analyzed the films' distinct color domains to reveal the relationship between the size distribution of the particles, phase transition onset, and deposition rate. For this purpose, the CNC films were flaked from the substrate and redispersed in Milli‐Q water. The size distribution of the CNCs in these different color zones was analyzed by AFM image analysis by depositing them on a mica substrate from redispersed suspensions. **Figure** **6**a,b confirmed that the blue color zone presents the CNCs with longer length at 277 ± 78 nm, compared to the green zone with 142 ± 51 nm and red zone with 117 ± 53 nm, and the height profiling analysis of the short axis of CNC is presented in Figure S14 (Supporting Information). As the color zones progress through a redshift, the aspect ratio decreases correspondingly from 27.2 in the blue zone to 22.1 in the green zone and finally to 14.8 in the red zone, as shown in Figure 6c. Our previous work elucidating the relationship between the particle aspect ratio, the gelation characteristics, and the final color of the CNC films established that the higher aspect ratio particles have an earlier onset for the phase transition and yield blue coloration, whereas the suspensions with smaller aspect ratio CNCs yielded a red shift in the coloration.^[^ ^42^ ^]^ While particle size distribution can be tuned using differential centrifugation to achieve color tuning, sonication and electrolyte addition strategies also allow manipulating the reflective properties.^[^ ^49^ ^]^ In these prior works, the efforts were primarily focused on tuning the particle size distribution and optimizing the electrostatic interactions to modify the final coloration of the CNC films. In contrast to those efforts, our work demonstrates, for the first time, that the different particle size distributions within a polydisperse suspension can also form distinct color gradients in a single‐step self‐assembly process.

A plausible explanation for the formation of distinct color zones at various stages of self‐assembly is the early onset of the phase transition in particles with a higher aspect ratio. This aligns with Onsager's theory, predicting nematic and cholesteric phase transitions to take place at critical volume concentrations (φ) at φ_i_ = 3.3 d/L and φ_a_ = 4.5 d/L, respectively where d and L are particle diameter and length.^[^ ^6^, ^50^ ^]^ According to the theory, the higher aspect ratio particles should have an earlier onset of phase transition. Therefore, in the angular deposition setup, in a polydisperse CNC suspension, the evaporation of water would cause selective segregation of the particles where the larger aspect ratio CNCs form tactoids at the earlier stages of evaporation.^[^ ^41^, ^51^ ^]^ These compact clusters experience stronger gravitational forces, causing them to sediment toward the container's base.^[^ ^24^, ^52^ ^]^ By contrast, CNCs with smaller aspect ratios would begin to agglomerate into tactoids at later stages of the self‐assembly process. Therefore at the earlier stages of the evaporation and self‐assembly process, it is expected that the small aspect ratio particles will suspend toward the top of the vial. The gravity‐driven sedimentation of CNC tactoids also creates a concentration gradient within the suspension from smaller particles at the top, and larger particles and pre‐agglomerates toward the bottom. Once the deposition process takes place in the meniscus area, the least dense tactoids from smaller aspect ratio particles are expected to be first transported by the capillary and Marangoni flow and deposited in the top region of the substrate. This is followed by tactoids of intermediate density accumulating in the middle region and the densest settling along the bottom edge as the drying process progresses. This gravity‐driven size distribution of the tactoids and the contribution from the Marangoni and capillary flows explain the color distribution of photonic films produced using the angular deposition setup.

## 3 Conclusion

In summary, we demonstrated the fabrication of photonic cellulose films with a color gradient in an angular deposition setup. The microstructural and optical analysis of the films revealed the mechanisms governing the alignment and orientation of CNC tactoid domains as well as their distribution across the self‐assembled film. This highlighted the significant role of the deposition angle influencing the self‐assembly behavior of CNC rods and the trade‐off between the capillary flow, Marangoni flow and gravity fields. Three distinct CNC domain arrangements, i.e., folding domains, misaligned domains, and well‐aligned domains, emerged sequentially on the substrate at the different stages of self‐assembly.

The microstructural analysis of the films provided insights into the effect of flow dynamics in the meniscus region concerning the self‐assembly of CNC particles by guiding the migration of tactoids and their subsequent interaction with the substrate during the film deposition process. Near the meniscus, during the initial stage of the deposition process, Marangoni flows cause tilting of the tactoids, which are then kinetically arrested in place as gelation occurs. In the later stages, we concluded that the reduced Marangoni flow was insufficient to counter‐balance the gravitational field, leading to misaligned domains. Such distinct tilt domains can modify the effective pitch and the twist of the tactoids helix, causing redshifts in the reflected wavelength and the reflection of the light with a mixture of polarization states. At the final stages of the self‐assembly, where gelation and slower evaporation promote strong vertical alignment due to the combined effect of capillary, Marangoni, and gravitational forces, we observed the formation of the well‐aligned domains.

We further quantitatively corroborated the gravity‐dependent sedimentation of CNC tactoids as a key factor in the formation of color zones on the substrate. Our analysis revealed that CNC size variation plays a critical role in determining the color zone formation during self‐assembly. This study is the first to use an angular deposition setup to manipulate the tilt domains of CNC tactoids and propose the mechanism for their formation. One thing to bear in mind is that the deposition of the particles and the balance between the flows and force gradients can also be manipulated via changing the wetting properties of the substrate.

Our insights into the control and understanding of the orientation and tilt of domains are crucial for optimizing the performance of CNC‐based optical materials and devices. Further studies on the simulation of flow dynamics in the meniscus area during angular self‐assembly, and CNC particle size fractionation will offer valuable insights to achieve precise color control in CNC films through the angular self‐assembly process.

## 4 Experimental Section

### Materials

Concentrated sulfuric acid (98 wt.%) and Whatman No1 filter paper were purchased from Sigma‐Aldrich Company Co., Ltd. (UK). Dialysis tubing (Mw cutoff 14000 kDa) was purchased from Thermo Fisher Scientific (UK). Milli‐Q water (18 MΩ cm^−1^ resistivity) was supplied from a molecular water purification system, Purite Fusion 160 (SUEZ).

### Extraction of Cellulose Nanocrystals

CNCs were extracted from filter paper by acid hydrolysis through following the method optimized by Dumanli et al.^[^ ^9^, ^19^ ^]^ Briefly, 10 g of filter paper was first shredded using a paper shredder and dissolved in 150 mL of sulfuric acid (64 wt.%) at 50 °C under vigorous stirring for 3 h. The cellulose‐acid slurry was quenched in 1.5 L of iced deionized water to terminate the hydrolysis reaction and allow it to settle overnight. The supernatant layer was then removed, and the remaining suspension was washed with Milli‐Q water several times, and each time the supernatant was removed via centrifugation (5000 rpm). The stable translucent‐white suspension obtained after the last centrifugation step was then placed inside the dialysis membrane tubes with a 12 000–14 000 MW cut‐off and dialyzed against water for 1–2 weeks until the pH of the CNC suspension reached between 6 and 7. This final suspension was concentrated by rotary evaporator to reach a concentration of 2 wt.%. To prepare the CNCs of different size distribution, the CNC stock suspension was treated with a tip‐sonicator (UP400S, Hielscher Ultrasonics) at 80% amplitude strength for 0, 3, and 6 min to obtain CNCs with larger, medium, and small aspect ratio. These tip‐sonicated CNCs were utilized to test the effect of size distribution on the color appearance of CNC films after self‐assembly.

### Zeta Potential Analysis and Size Distribution of the CNCs

The particle size, i.e., hydrodynamic diameter (D_h_), was measured using a Malvern Zetasizer Ultra (laser wavelength of 633 nm (He–Ne), scattering angle 173 °, medium viscosity 0.8872 mPa s, medium refractive index 1.330) DLS system. Before the measurements, all samples were treated with the ultra‐sonication bath for 10 min to ensure the CNC particles were freely suspended. The Z‐average diameter was recorded using a thermal equilibration time of 120 s in a folded capillary cell (DTS1070, Malvern). Capillary cells were flushed with ethanol and water prior to usage. Measurements were repeated in triplicate to give a mean Z‐average diameter and polydispersity index (PdI).

Atomic force microscopy (AFM, Bruker Multimode 8) was employed to verify the DLS measurements in Scanasyst air mode with scanasyst‐air probe (tip radius: 2 nm, spring constant: 0.4 N m^−1^, frequency: 70 kHz). Prior to imaging, a droplet of CNCs suspension (5 µL, 0.001 wt.%) was cast on a mica substrate (5 × 5 mm). The droplets were allowed to dry under ambient conditions overnight. CNC films of distinct color domains were flaked from the substrate and redispersed in Milli‐Q water under a sonication bath for 3 h. The size distribution of the CNCs in these different color zones was analyzed by AFM image analysis by depositing them on a mica substrate from redispersed suspensions.

### Self‐Assembly of CNCs at Angular Deposition

The angular deposition setup was designed by attaching the container (polystyrene cuvettes, DTS0012, Malvern) to a custom‐made holder with a well‐defined deposition angle. In this setup, 2 mL of the CNC suspensions (2 wt.%) without macroscopic phase separation was used for self‐assembly. The incubation conditions for angular deposition were kept at 60 °C, 20% RH using a Memmert Humidity cabinet (HPP110). The samples obtained in this experiment were denoted as CNC‐θ, where θ indicates the deposition angle. To compare with the angularly deposited film, a drop‐casted CNC film was prepared by placing 0.5 mL of CNC suspension (2 wt.%) within a circular boundary (16 mm diameter) confined by tape.

### Height Profile Analysis

The thickness of the CNC film was measured by scanning across the sample with a DektakXT Stylus Profiler (Bruker) in the standard scan mode. A 2 µm radius stylus was used at a force of 5 mg, with a resolution of 0.33 µm and a speed of 100 µm s^−1^. During the measurement, the films were positioned on a flat glass slide.

### Optical and Spectral Analysis

The optical imaging and spectroscopy measurements were performed using a custom‐modified BX‐53 Olympus optical microscope equipped with a color digital CCD camera (Lumenera, Infinity 1–3C). Light from an LED lamp was coupled into a 20× (Olympus, LMPLFLN‐BD 20 NA = 0.3). The reflected signal from the sample was observed in unpolarized, cross‐polarized modes using the microscope optics. A custom add‐on feature was built from a 3‐d printed enclosure, a commercial slider, and polymeric quarter wave‐plate polarizers to allow left and right‐polarized light observations (ELL9K, CP1R532, and CP1L532, respectively. Thorlabs). For the spectral analysis, the microscope is designed with a switch allowing to change of the light path to be direct to a UV–vis spectrometer (FLAME, Ocean Insight). In the spectrometer configuration, the transmitted signal was coupled into a 50‐µm core optical fiber (Ocean Insight) mounted in a confocal configuration to achieve a spot size of ≈10 µm. Circular dichroism spectra were recorded using an Applied Photophysics Chirascan Plus CD spectropolarimeter.

### Computer‐Assisted Image Analysis

Computer‐assisted image analysis was performed to improve the comparability of the samples in each group and thus quantify the distribution of colors. The color information was extracted from micrographs using the OpenCV, Color, NumPy, matplotlib, and Scikit‐learn libraries. The code was written in a Jupyter Notebook in Visual Studio Code using Python 3.11. Low‐intensity areas were first removed from micrographs via grayscale thresholding and mapping onto the original images. RGB data was then extracted and averaged from each micrograph using K‐Means clustering and plotted onto a 2‐d chart. Image J was used to quantitatively analyze the contribution of the RGB channel to the overall color appearance of CNC film and to measure the proportion of the colored area within the CNC film.

### Microstructural Analysis

The microstructure and morphology of the samples were characterized using a field emission scanning electron microscope (TESCAN MIRA II). The CNC films produced at varying deposition angles were fractured in half along the longitudinal direction to expose the cross‐section of the films. The cleaved samples were then attached on 90° angled SEM stubs (Agar‐Scientific, 45/90° chamfer) so that the cross‐section could be imaged. To prevent charging in the SEM, the samples were coated with ≈6 nm of Au/Pd in a sputter coater (Q150R Plus, Quorum) at a current of 40 mA.

## Conflict of Interest

The authors declare no conflict of interest.

## Author Contributions

Y.Z. designed, performed, and analyzed the experimental work on the self‐assembly of CNCs via angular deposition set‐up. Tadeusz Balcerowski performed the coding program, which is used for the computer‐assisted image analysis on the CNC films. A.G.D. supervised the project and discussions leading to the design of the manuscript. Y.Z. and A.G.D. drafted and made the initial iterations of the manuscript, and all the authors equally contributed to the manuscript review & editing process.

## Supporting information



Supporting Information

## Data Availability

The data that support the findings of this study are available from the corresponding author upon reasonable request.
