# Supplementary material for: Marangoni Flow‐Driven Angular Self‐Assembly of Cellulose Nanocrystals: The Tale of Tilted Tactoids and Folded Domains
Source: Small Methods. 2025 May 19;9(7):2401966. doi: 10.1002/smtd.202401966 (PMC12285622; doi:10.1002/smtd.202401966)
Supplement: Supplementary file 1 — Supporting Information [file SMTD-9-2401966-s001.docx]

**Marangoni flow-driven angular self-assembly of cellulose nanocrystals: The tale of tilted tactoids and folded domains**

Yuchen Zhu^1,2^, Tadeusz Balcerowski^1,2^ and Ahu Gümrah Dumanli^1,2*^

1 Department of Materials, The University of Manchester, Oxford Rd, Manchester, M13 9PL, UK

2 Henry Royce Institute, The University of Manchester, Oxford Rd, Manchester, M13 9PL, UK

**Corresponding author email:**

**ahugumrah.parry@manchester.ac.uk**

**Supplementary data**

The CNCs were extracted from filter paper by modifying our previously reported method^[1,2]^. The particle size distribution and the colloidal stability of the CNCs obtained from sulfuric acid hydrolysis were tested using Atomic Force Microscopy (AFM), Dynamic Light Scattering (DLS) and zeta-potential measurements. From the AFM measurements, the particles had an average length distribution between 120 nm and 180 nm, and the zeta-potential at -48 mV indicated quite robust colloidal stability. Fig. S1 presents the results of size distribution and zeta potential of CNCs by DLS, AFM and zeta potential analyser.


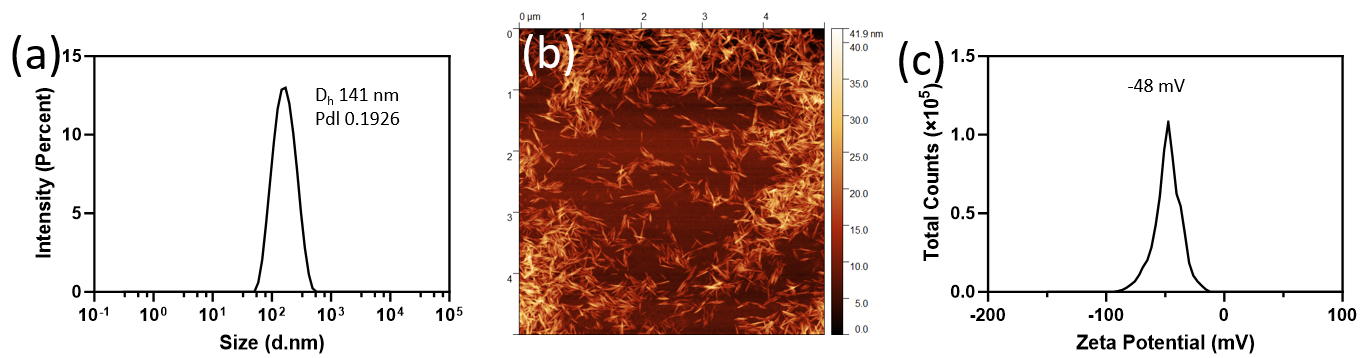


Fig. S1. Size distribution of CNCs measured by (a) DLS and (b) AFM, and (c) the distribution profile of their zeta potential.


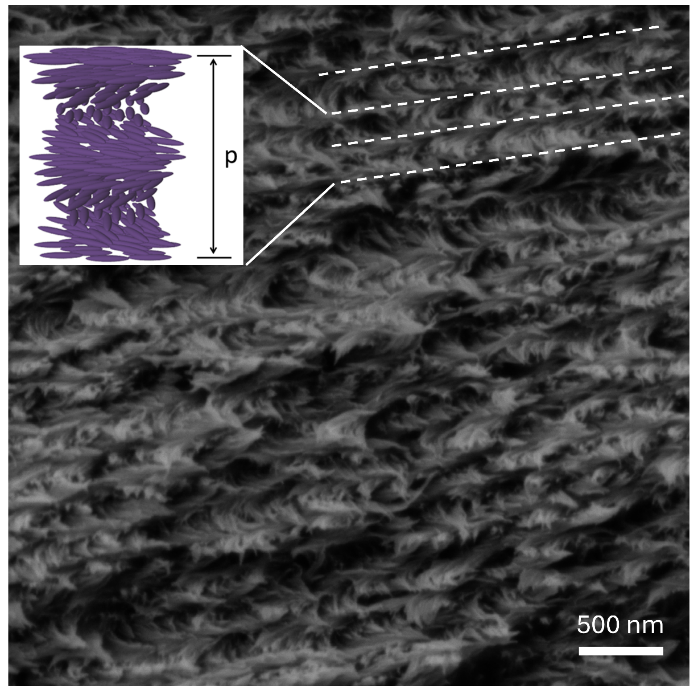


Fig. S2. High resolution SEM image of the CNC film showing a left-handed helical structure.


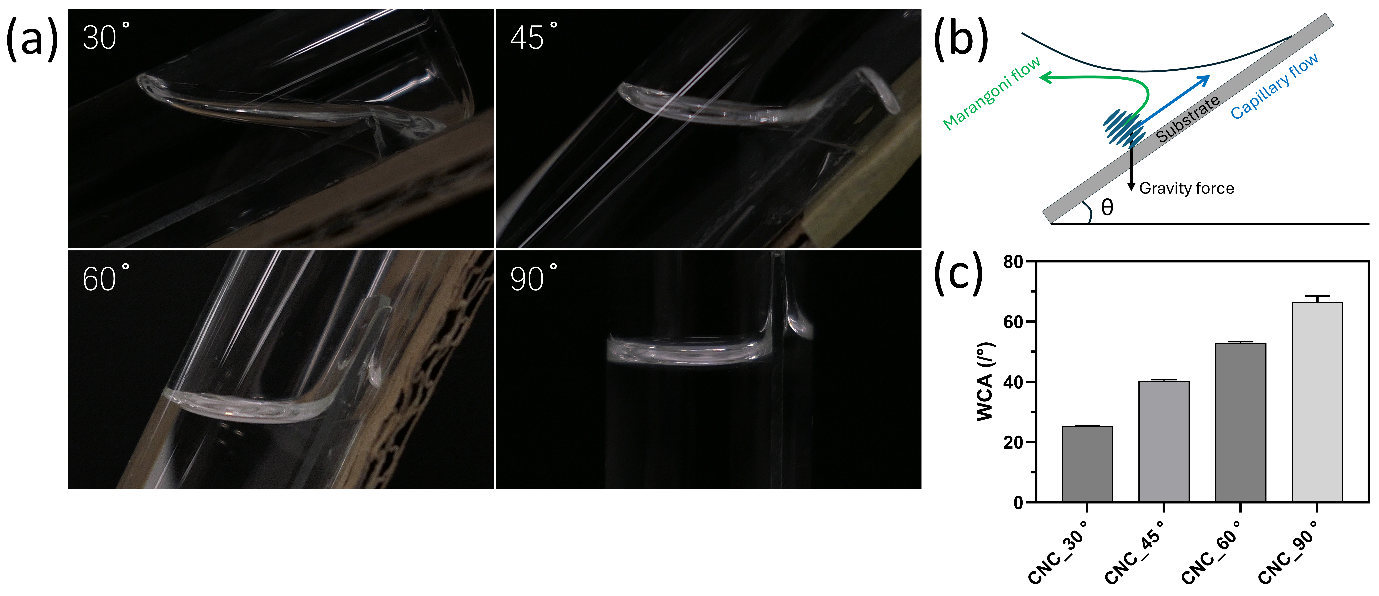


Fig. S3. (a) Digital photographs of angular setup at the specific deposition angle; (b) Sketch of force balance by the Marangoni flow, capillary flow and gravity force; (c) water contact angle analysis of the meniscus at the contact line.


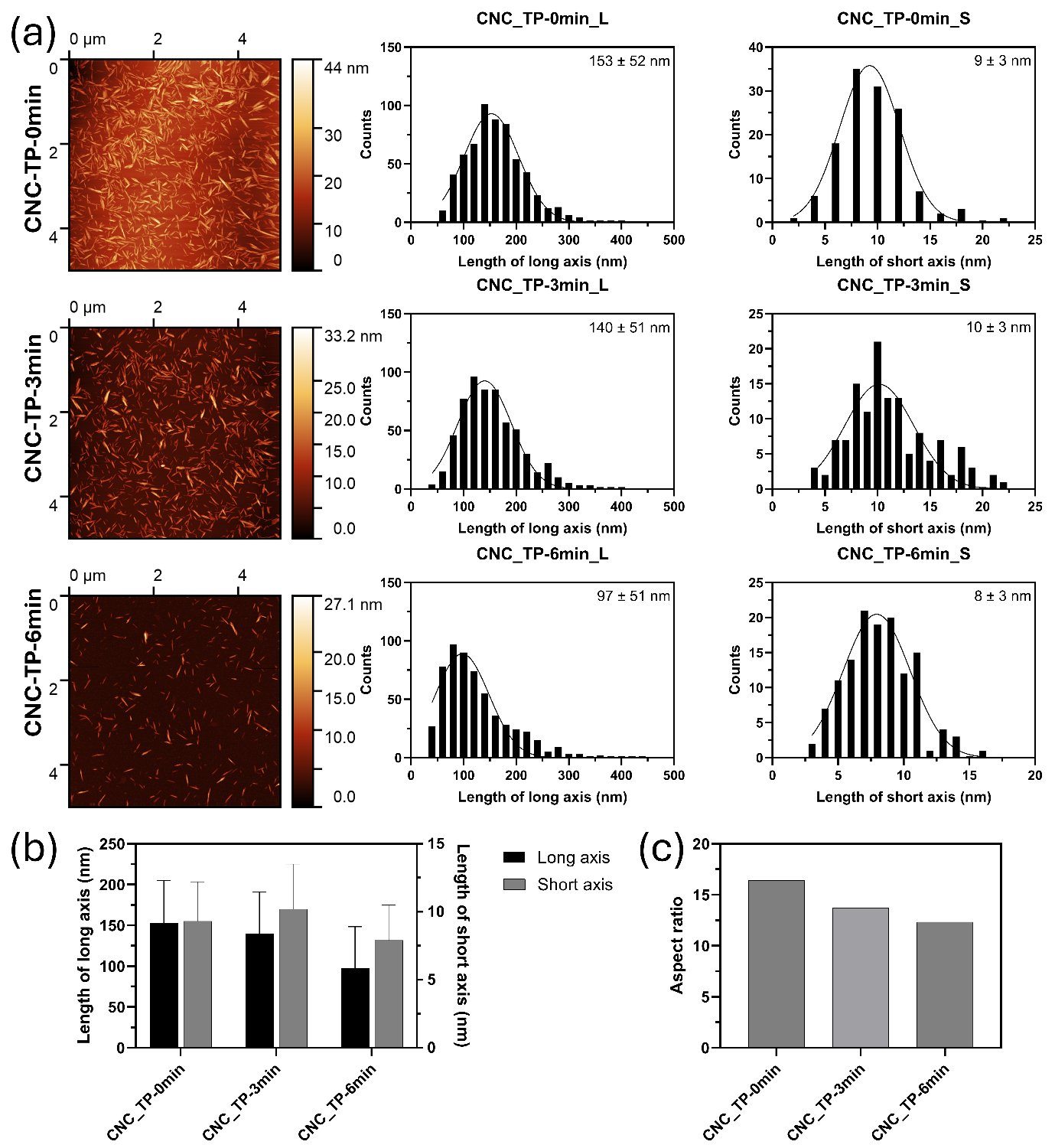


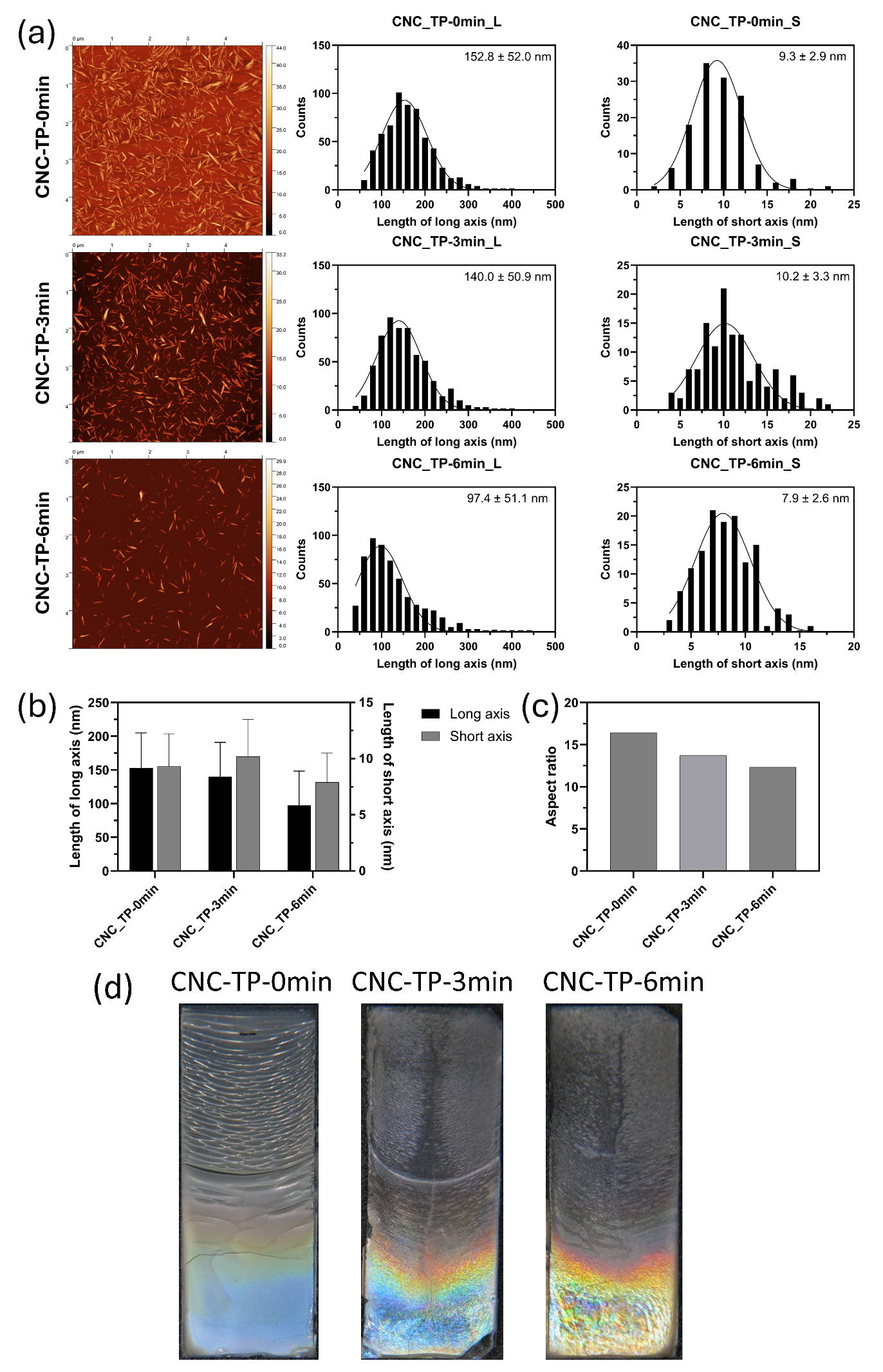


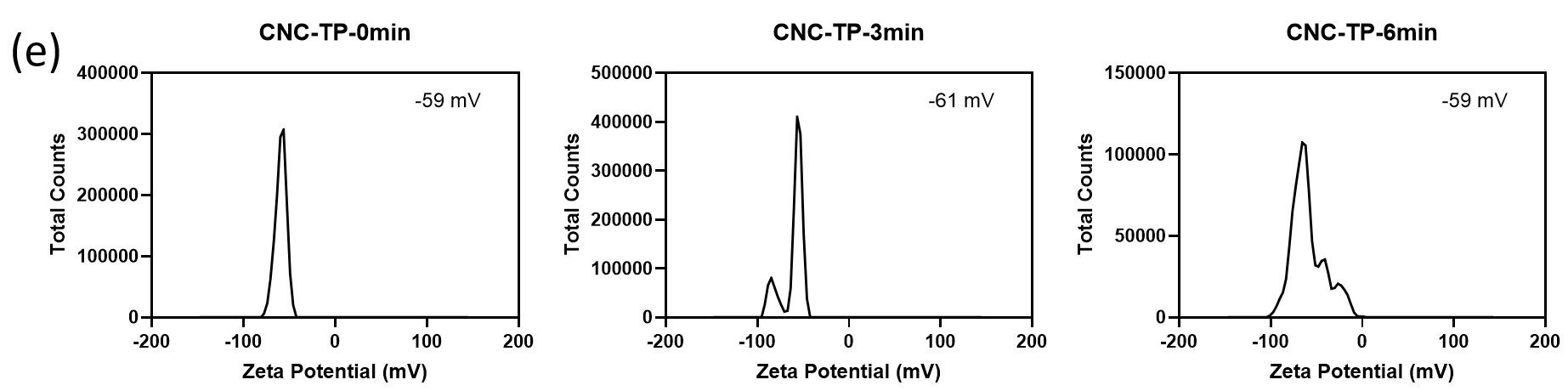


Fig. S4. (a) AFM images of the CNCs subjected to tip sonication for 0 min (CNC-TP-0min), for 3 min (CNC-TP-3min), and for 6 min (CNC-TP-6min) and (b) size distribution of the tip sonicated suspension for length determination of the long axis and the short axis, (c) aspect ratio analysis of tip sonicated CNC particles (d) CNC photonic film fabricated from tip sonicated suspension (e) Zeta potential distribution of CNC particles treated with the tip sonication for 0 min, 3 min, and 6 min.


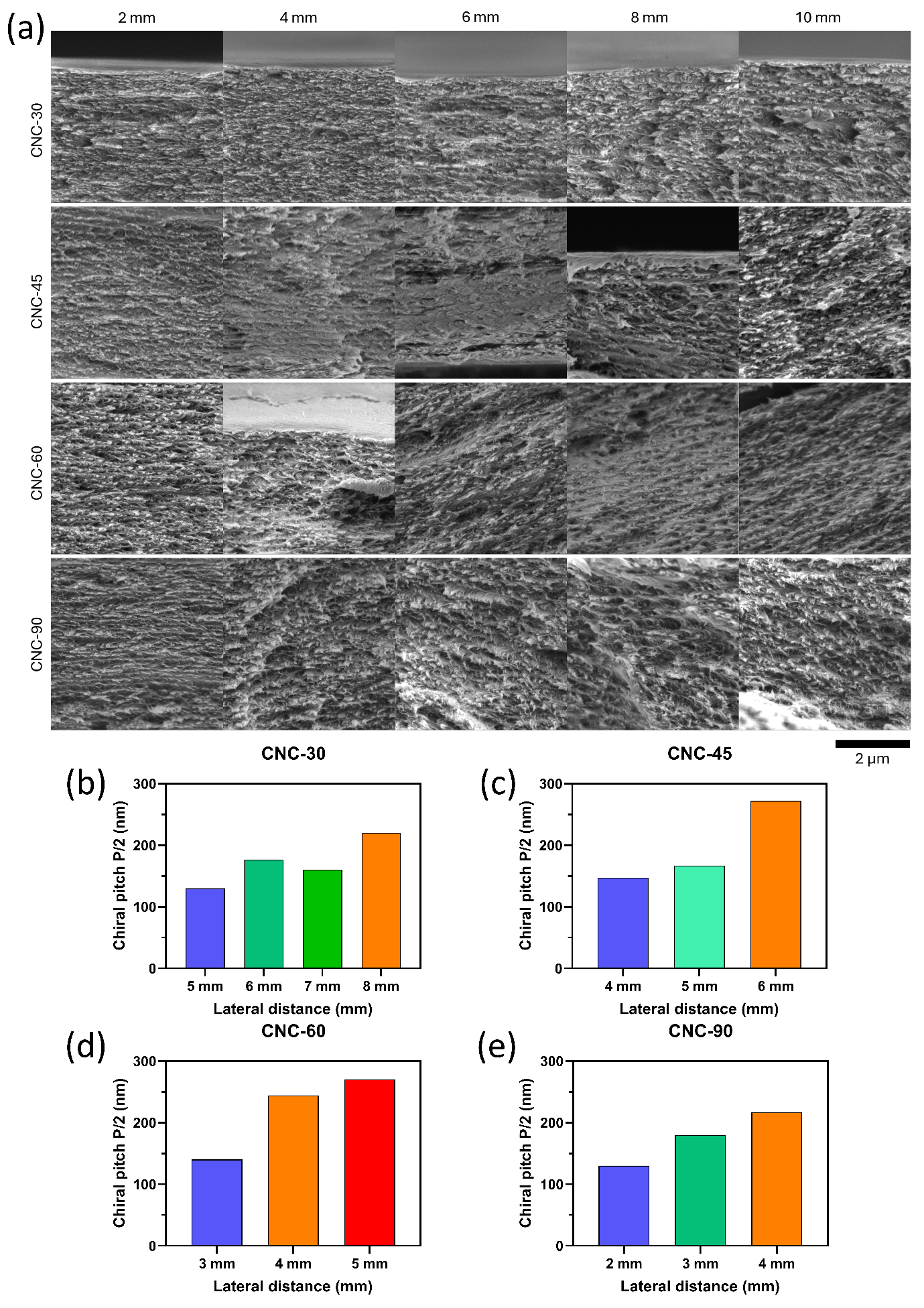


Fig. S5. (a) Cross-sectional SEM images of CNC films analysed at 2, 4, 6, 8, and 10 mm marking from the bottom of the glass slide toward the top. (b), (c), (d), and (e) chiral pitch analysis extracted from CNC-30, CNC-45, CNC-60, and CNC-90 film, respectively. The scale bar is 2 µm.


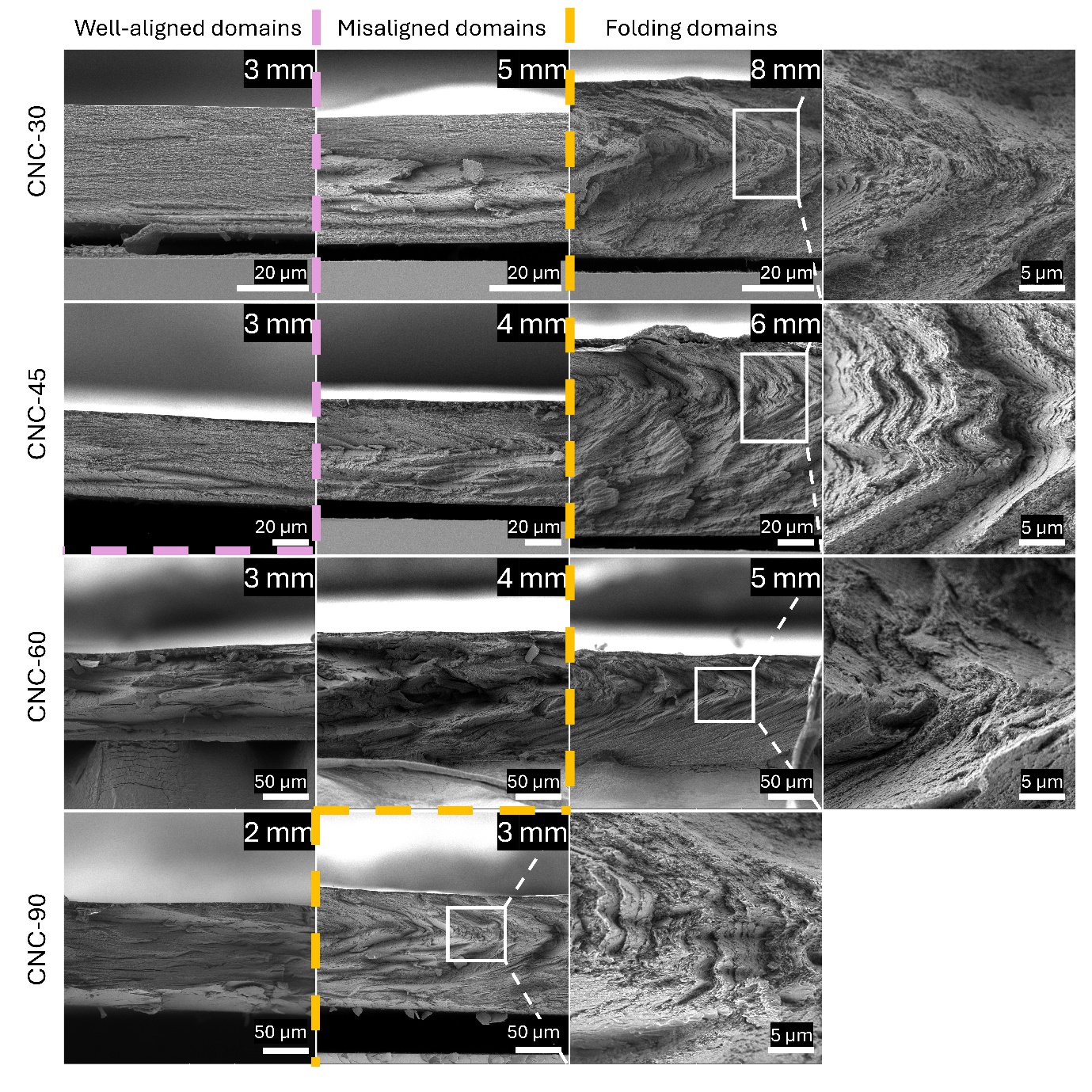


**Fig. S6. Representative SEM images of multi-hierarchical microstructure of CNC tactoid domains** **at different structural transition stage.**


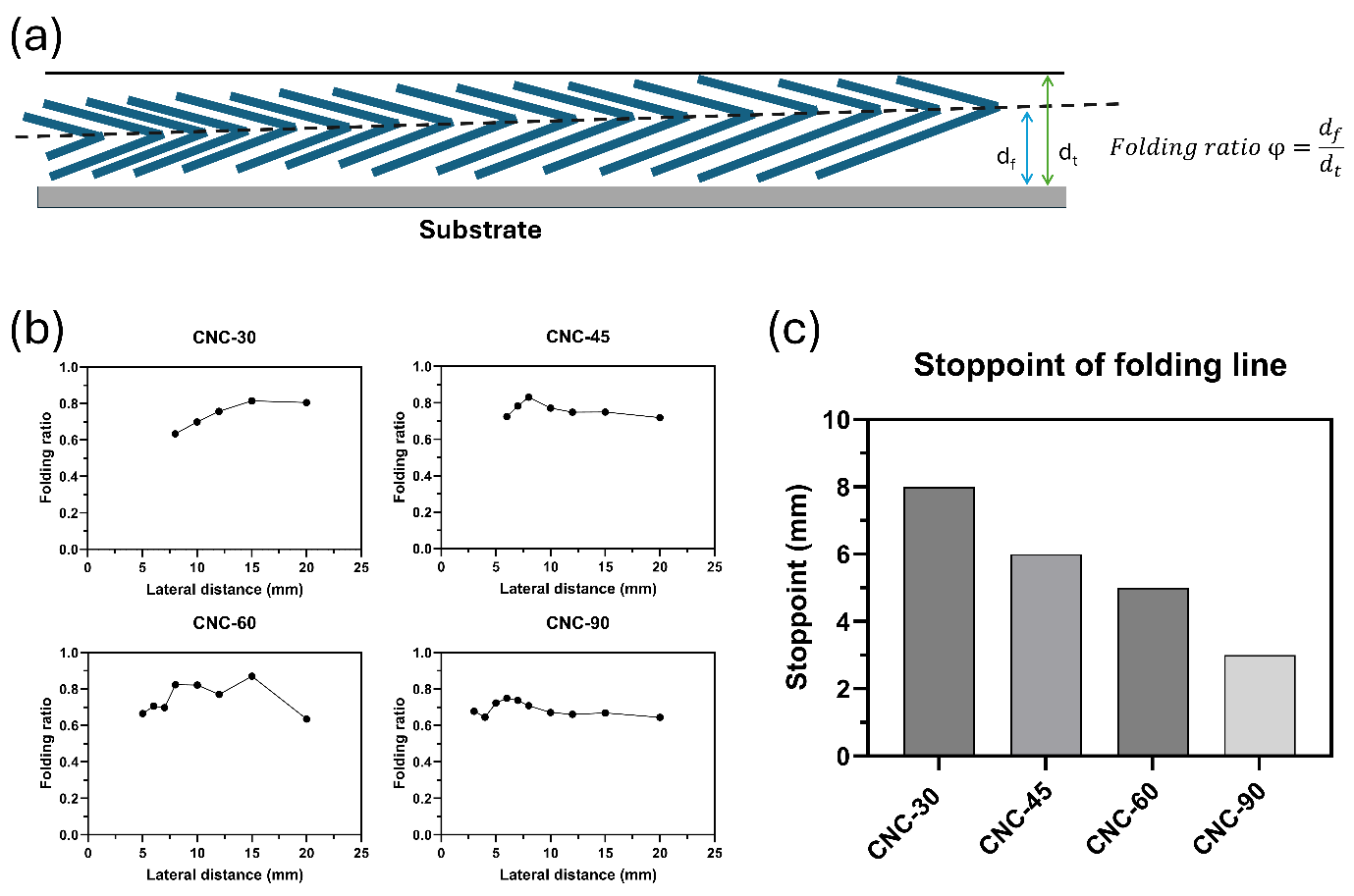


**Fig. S7. Schematic diagram for the formation of CNC folding domains.** (a) Schematic representative of the formation of the folding line; (b) The folding ratio of samples at different deposition angles, (c) Stoppoint analysis of tactoid folding line.

During the final stages of evaporation in angular depositions, the cholesteric structure experiences a uniaxial compression along the surface normal, instead of uniform compression in all direction. This leads to varying compression efficiencies for the domains tilted at different angles. The anisotropic compression applied on the tilt domains can be adapted from the formula derived from Bruno’s publications^[3–6]^. According to this formula, the compressed pitch, p´, can be expressed in terms of the original pitch p, compression factor α, and final tilt angle β by the below equation (1).

$p^{'}=p\sqrt{{sin}^{2}\beta+\alpha^{2}{cos}^{2}\beta}$ (1)

This equation illustrates how the pitch of CNC tactoids changes against the tilt angle following vertical compression, as shown in the schematic diagram below:


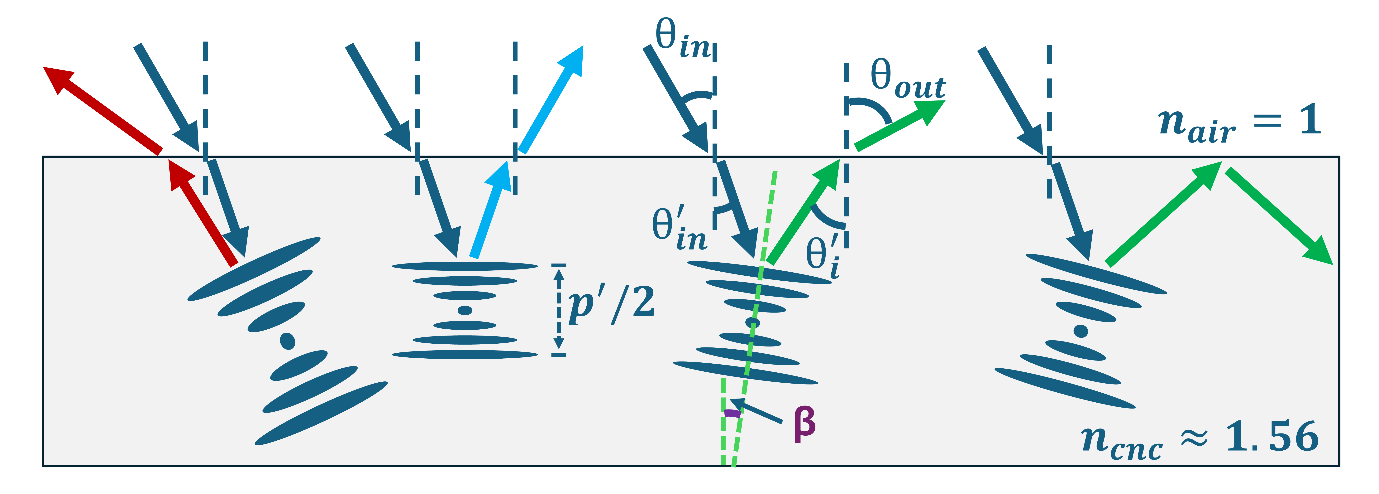


Fig. S8. Schematic diagram of tactoids at different alignment with respect to specular reflectance point of view.

When light is incident on the CNC at a certain angle, it is refracted at the film-air interface before reaching the CNC tactoids. This incident angle ${}_{in}^{'}$.in CNC films can be determined using Snell’s law. The wavelength of peak reflection for the tilted cholesteric structure is then calculated using the Bragg equation, as shown below:

$\lambda_{max}=np^{'}cos\left\{ \frac{1}{2}\left[ {sin}^{-1}\left( \frac{{sin}_{out}}{n_{cnc}} \right)-{sin}^{-1}\left( \frac{{sin}_{in}}{n_{cnc}} \right) \right] \right\}$ (2)

where $n_{cnc}$ is the average refractive index of the CNC film, ${}_{in}$ is the incident angle of light, ${}_{out}$ is the outgoing angle of light from CNC film.


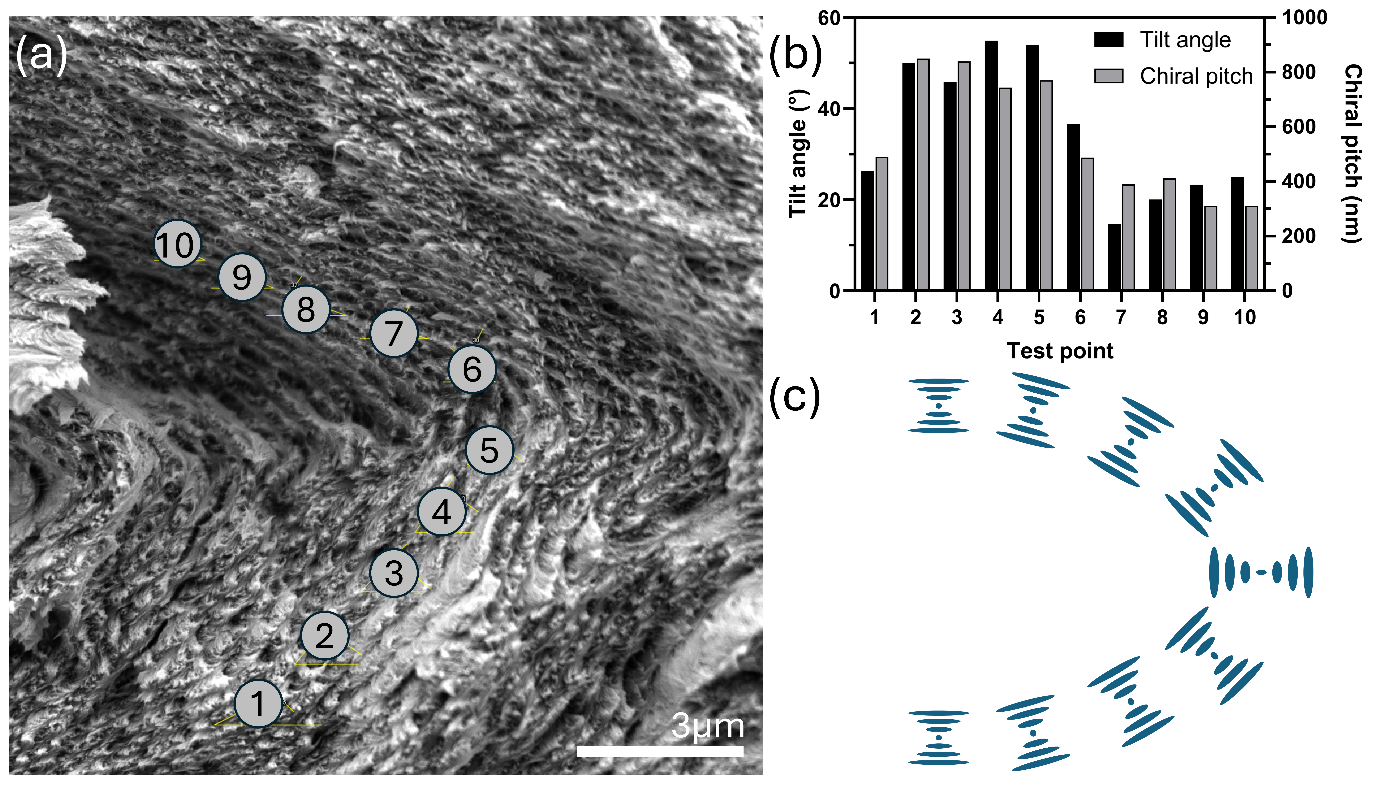


Fig. S9. SEM image of the cross-section of CNC-90 film with (a) folding domain and (b) its pitch variation analysis from the point 1 to 10, (c) schematic representation of the pitch under compression along the folding domain.


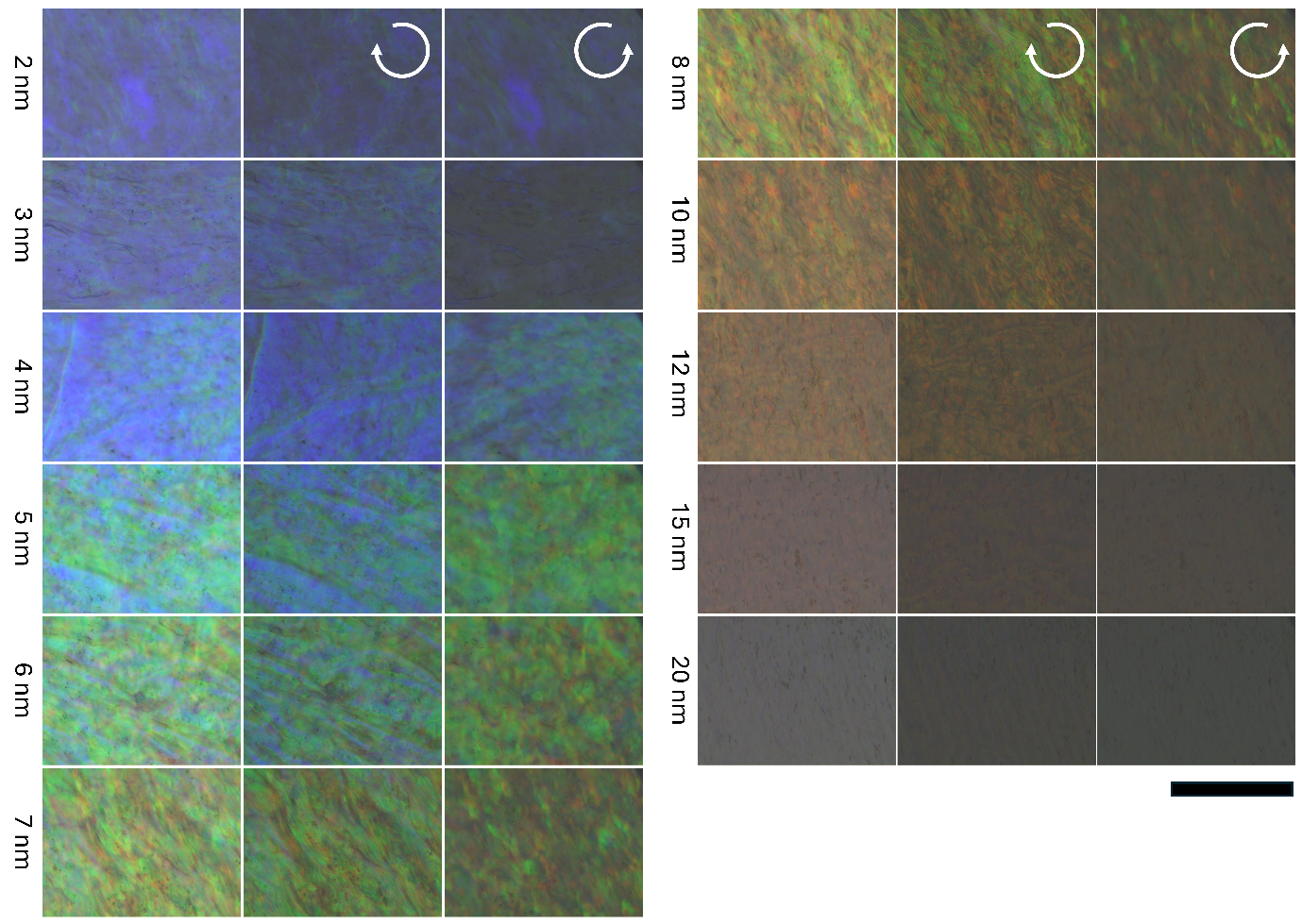


Fig. S10. Optical microscopy images of CNC-30 film under linear, circular left, and circular right polarisation at analysis points. The scale bar is 200 µm.


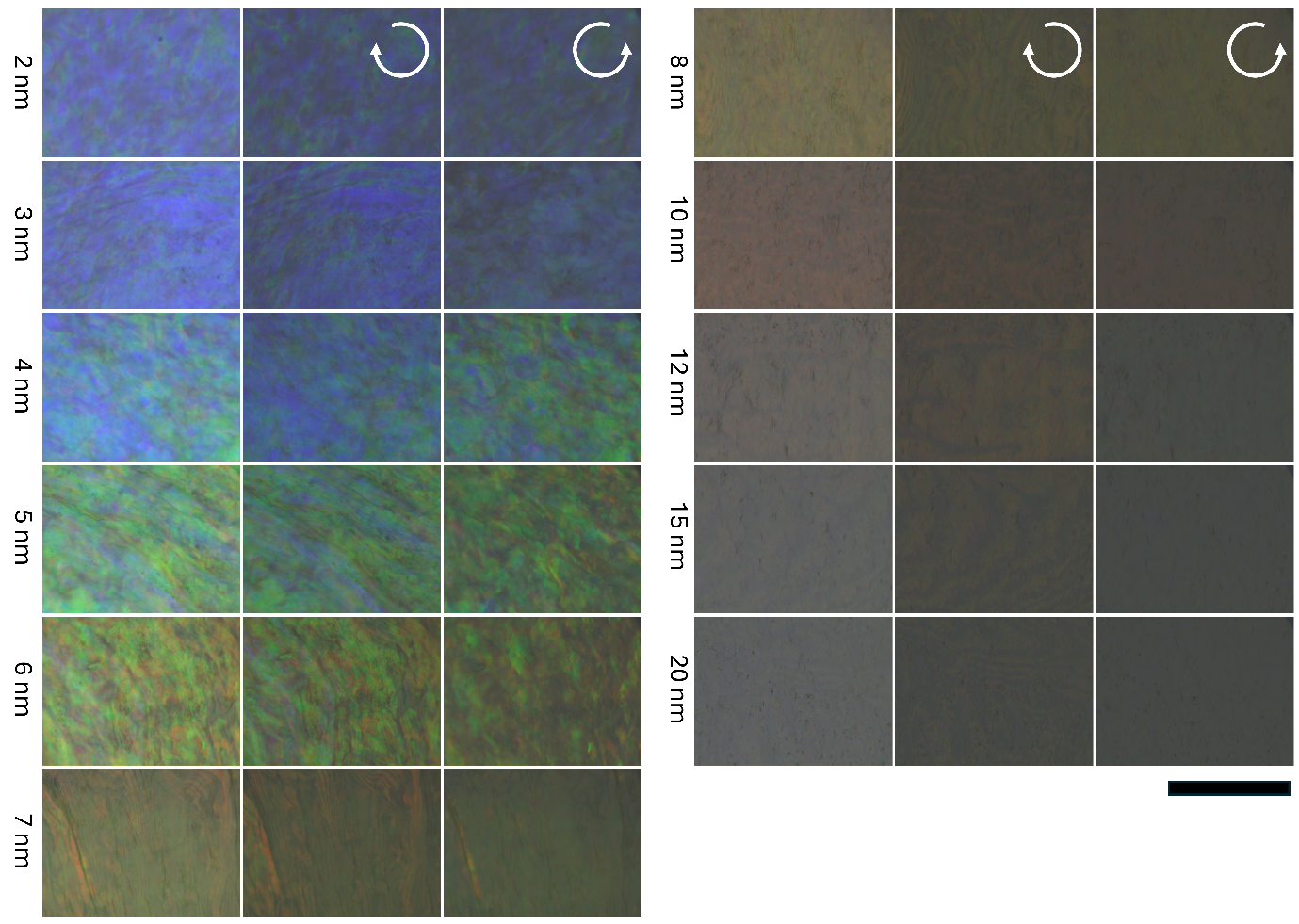


Fig. S11. Optical microscopy images of CNC-45 film under linear, circular left, and circular right polarisation at analysis points. The scale bar is 200 µm.


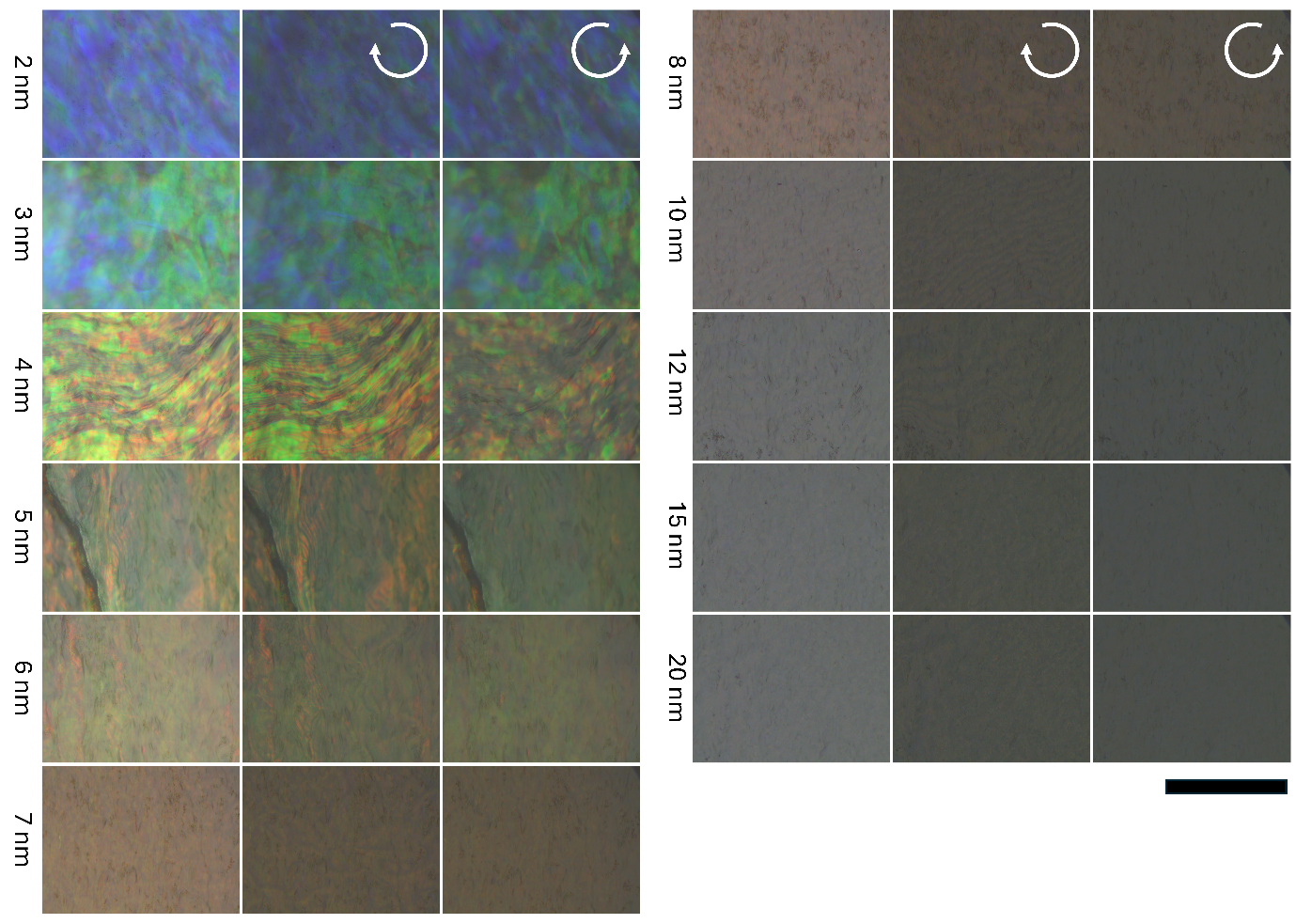


Fig. S12. Optical microscopy images of CNC-60 film under linear, circular left, and circular right polarisation at analysis points. The scale bar is 200 µm.


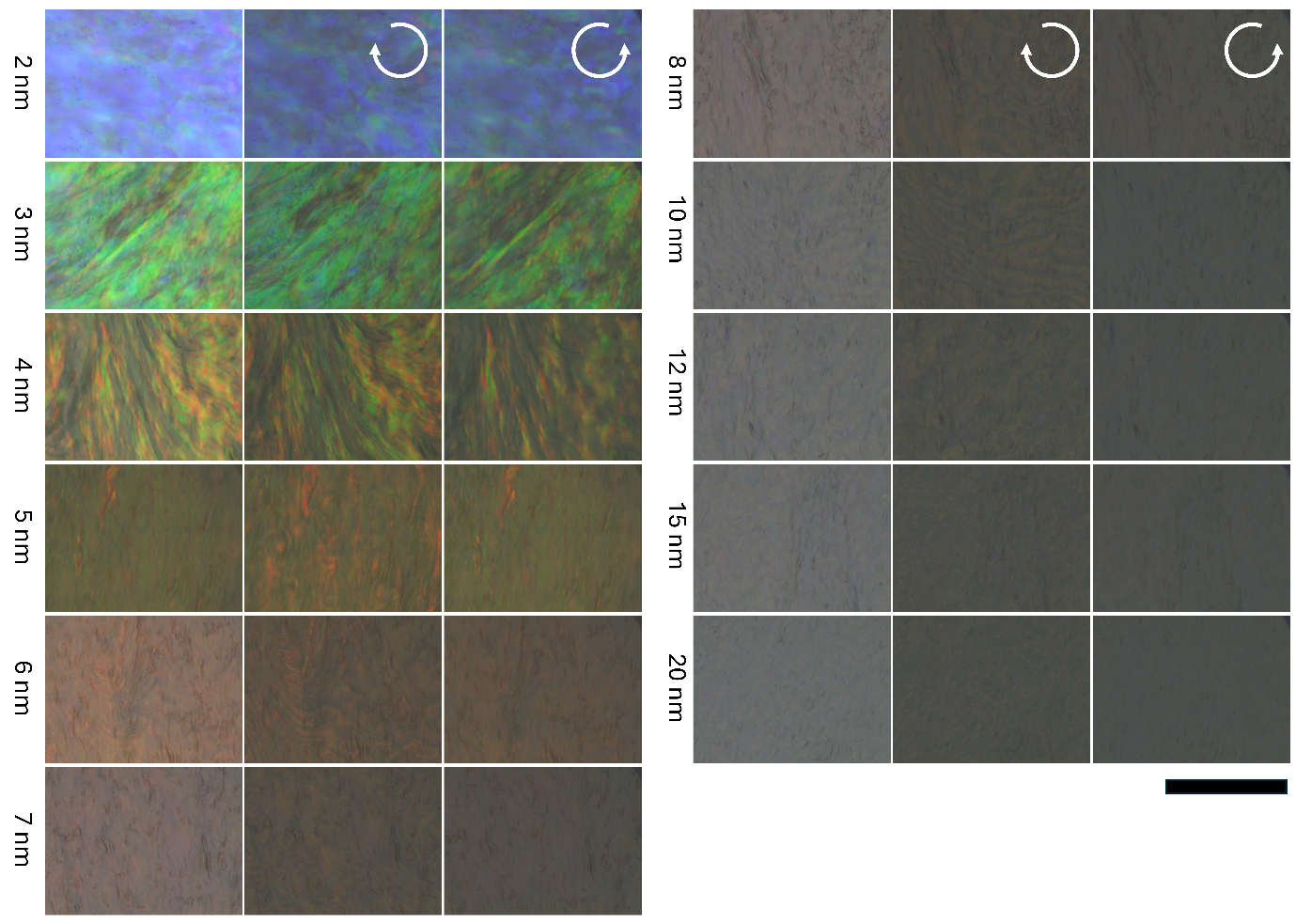


Fig. S13. Optical microscopy images of CNC-90 film under linear, circular left, and circular right polarisation at analysis points. The scale bar is 200 µm.


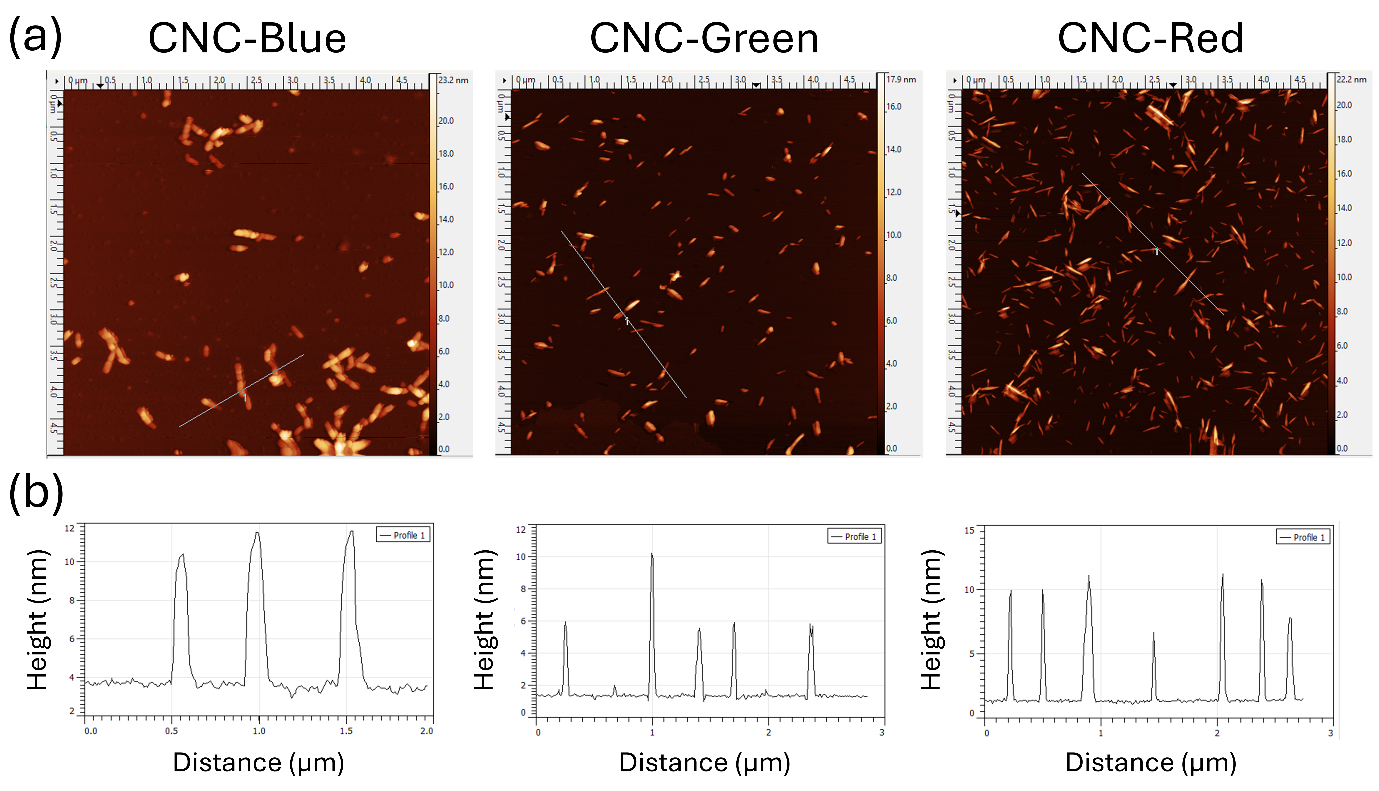


Fig. S14. (a) AFM images of CNC particles extracted from blue, green, and red zone of CNC film and their (b) corresponding height profile.

**Reference**

[1] A. G. Dumanli, H. M. Van Der Kooij, G. Kamita, E. Reisner, J. J. Baumberg, U. Steiner, S. Vignolini, *ACS Appl Mater Interfaces* 2014, *6*, 12302.

[2] A. G. Dumanli, G. Kamita, J. Landman, H. van der Kooij, B. J. Glover, J. J. Baumberg, U. Steiner, S. Vignolini, *Adv Opt Mater* 2014, *2*, 646.

[3] B. Frka-Petesic, J. A. Kelly, G. Jacucci, G. Guidetti, G. Kamita, N. P. Crossette, W. Y. Hamad, M. J. MacLachlan, S. Vignolini, *Advanced Materials* 2020, *32*, DOI 10.1002/adma.201906889.

[4] B. Frka-petesic, G. Guidetti, G. Kamita, S. Vignolini, 2017, *1701469*, 1.

[5] R. M. Parker, G. Guidetti, C. A. Williams, T. Zhao, A. Narkevicius, S. Vignolini, B. Frka-Petesic, *Advanced Materials* 2018, *30*, DOI 10.1002/adma.201704477.

[6] B. Frka-Petesic, G. Kamita, G. Guidetti, S. Vignolini, *Phys Rev Mater* 2019, *3*, 45601.
